# Lamin B receptor upregulation in metastatic melanoma causes nuclear envelope fragility in confined migration during cancer invasion

**DOI:** 10.1073/pnas.2513031123

**Published:** 2026-02-18

**Authors:** Michelle A. Baird, Cayla E. Jewett, Daniela A. Malide, Lisa Kratz, Alexander X. Cartagena-Rivera, Robert S. Fischer, Clare M. Waterman

**Affiliations:** ^a^Cell and Developmental Biology Center, National Heart, Lung and Blood Institute, NIH, Bethesda, MD 20892; ^b^Department of Cell and Developmental Biology, University of Colorado Anschutz Medical Campus, Aurora, CO 80045; ^c^National Heart, Lung and Blood Institute, NIH, Bethesda, MD 20892; ^d^Department of Genetic Medicine, Kennedy Krieger Institute, Johns Hopkins School of Medicine, Baltimore, MD 21205; ^e^Section on Mechanobiology, National Institute of Biomedical Imaging and Bioengineering, NIH, Bethesda, MD 20892

**Keywords:** nuclear envelope, nuclear rupture, metastasis, melanoma, nucleus

## Abstract

Cancer cells migrate through regions of tissue confinement during metastasis, causing nuclear envelope (NE) rupture, which generates heritable DNA damage and disease aggression. We sought to determine if NE rupture was intrinsic to malignant transformation and seek a mechanistic cause. We found that metastatic cells from multiple cancer subtypes have increased NE fragility in confinement compared to their benign counterparts. Meta-analysis of transcriptomic data from clinical samples of melanoma progression together with an siRNA-based live-cell confinement screen revealed that lamin B receptor (LBR) transcriptional upregulation correlates with melanoma progression and NE fragility. LBR is an inner nuclear membrane protein (INM) that scaffolds lamins and chromatin and has sterol reductase activity in the cholesterol biosynthesis pathway. Applying superresolution and atomic force microscopies to characterize the cellular and biophysical events leading to NE rupture revealed that upregulated LBR increased nuclear deformability and can generate local ruptures of the INM, promoting blebs in the nuclear membrane that burst to release nuclear contents into the cytoplasm. Structure–function analysis showed that LBR’s sterol reductase activity is required for its promotion of NE fragility in cells confined in vitro. Use of tumor organoids and an in vivo melanoma model revealed that upregulation of LBR was associated with increased NE fragility, metastatic invasion, and decreased patient survival. Thus, upregulation of LBR in melanoma promotes nuclear deformability, while LBR’s sterol reductase activity causes fragility and instability of the nuclear membrane, and these changes in the nucleus provide a possible mechanism for increased genetic heterogeneity in melanomas.

## Significance

To metastasize, cancer cells migrate from the primary tumor through dense tissues to spread to distant organs. During this journey, as cells squeeze through thin passages in tissues, the membrane surrounding the cancer cell’s nucleus can burst, causing DNA damage and mutations, making it more malignant. We found that a specific protein, lamin B receptor (LBR), gets overproduced in aggressive melanoma. We found in isolated cancer cells that excess LBR causes cell nuclei to become more deformable and fragile, making them susceptible to rupture and that LBR-induced nuclear fragility happens in skin tumors in mice, increases cancer spreading, and correlates with decreased melanoma patient survival. This identifies LBR as a potential therapeutic target in the prevention of melanoma metastasis.

Metastatic spread accounts for most cancer deaths, yet its mechanisms remain poorly understood (1). During metastasis, tumor cells migrate through regions of cellular confinement caused by dense tissue architecture (2), increased collagen bundling in the tumor microenvironment (3), and squeezing through vascular walls during transendothelial migration (4). While the cytoplasm deforms easily through the 1 to 30 µm pore size of such confined tissue regions (5), the nucleus is mechanically stiff, and its deformation during confined migration causes rupture of the nuclear envelope (NE) (6, 7). NE rupture exposes the genome to cytoplasmic exonucleases and mislocalizes DNA repair factors, leading to heritable DNA damage and chromothripsis (8–11). While abnormal nuclear morphology is a hallmark in cancer diagnosis (12), how this relates to NE fragility during oncogenic transformation remains unclear.

While some cancer cells exhibit NE fragility, it is unknown whether this is a universal property of transformation or if its underlying mechanisms are conserved. The NE is composed of two parallel membranes joined at nuclear pore complexes (NPC) and bridged by the LINC complex, connecting the NE to the cytoskeleton (13, 14). The outer nuclear membrane (ONM) is continuous with the endoplasmic reticulum (ER), while the inner nuclear membrane (INM) is enriched with integral membrane proteins tethering it to the lamin nucleoskeleton (lamina) and heterochromatin, thus organizing the genome (15). For DNA to be exposed to damage during confined migration, chromatin must breach the lamina, INM and ONM, interactions among which contribute to mechanical properties of the nucleus (7, 16). Indeed, NE rupture can result from perturbation of lamin levels or function (17), weak coupling of lamins or chromatin to the NE (18), or through actomyosin contractility and the cytoskeleton (19). However, it remains unclear if there are additional mechanisms regulating NE fragility and if they play a role in metastatic progression.

Malignant melanoma is an aggressive skin cancer that often becomes highly invasive and resistant to chemotherapies, resulting in lethality. Melanoma is generally initiated by transformation of melanocytes by the BRAF^V600E^ mutation, causing hyperactivation of the mitogen-activated protein kinase (MAPK) pathway, driving proliferation and migration (20). Disease progresses from benign nevi to radial and vertical growth phases (stages 1-2), invasion (stages 3-4), and ultimately metastasis (21). Invasion correlates with ECM remodeling, causing increasing cancer cell confinement via collagen IV bundling (22), where NE rupture has been observed (2, 6). Transcriptomic analysis of tumors indicates that melanoma is one of the most genetically diverse cancers, with heterogeneous metastatic subclones promoting chemotherapeutic resistance through the proliferation of drug-resistant cells (23). Thus, understanding the mechanisms promoting NE fragility in metastatic melanoma is vital to preventing its metastatic spread and genetic heterogeneity, to ultimately avert chemotherapeutic resistance.

We asked whether NE fragility and altered nuclear mechanics were intrinsic properties of malignant transformation, and if so, to address the mechanistic cause. Bioinformatics, an siRNA-based cell-confinement screen, atomic force microscopy (AFM), and structure function analysis identified lamin B receptor’s sterol reductase activity and cholesterol as promoters of NE fragility and deformability, while tumor organoid and animal melanoma tumor models showed that LBR drives tumor invasion and NE rupture in vivo.

## Results

### NE Genes Are Transcriptionally Altered during Cancer Progression Promoting NE Fragility.

**Fig. 1.**
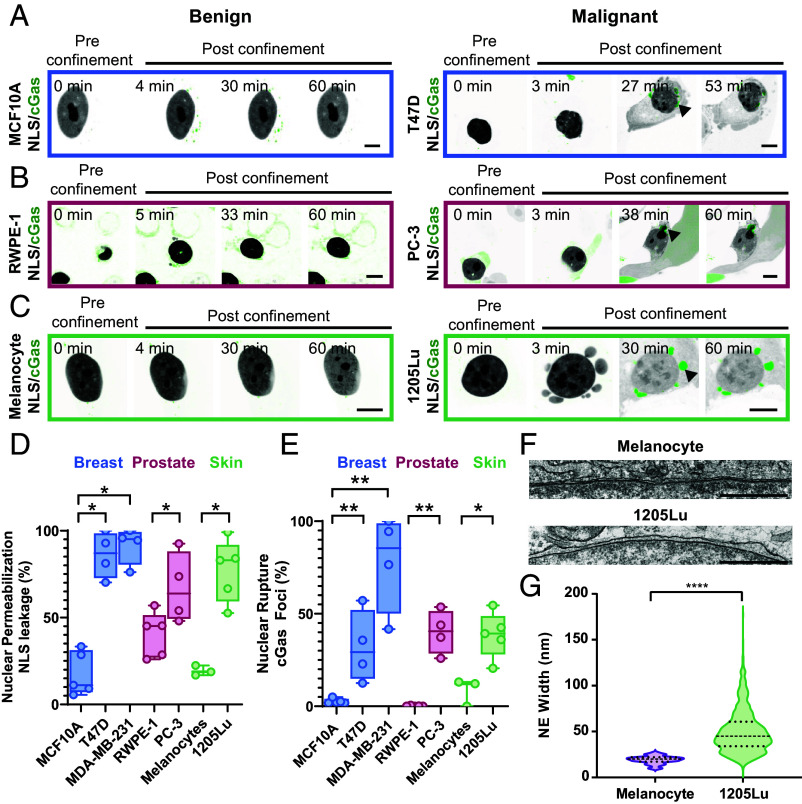
Cancer progression is associated with increased NE fragility under cellular confinement. (*A*–*C*) Confocal image series of benign (*Left*) or malignant (*Right*) cells (*A*) MCF10A or T47D (breast cells, blue); (*B*) RWPE-1 or PC-3 (prostate cells; rose outline); (*C*) melanocytes or 1205Lu (skin cells, green), with mCherry-NLS (inverted grayscale) and GFP-cGas (green) before (pre confinement) and after (post confinement) confinement to 3 µm. Arrows, perinuclear GFP-cGas foci, imaging timing noted. (*D* and *E*) Fraction of confined cells exhibiting NE permeabilization (*Left*) or NE rupture (*Right*), from benign and malignant breast (blue, MCF10A (n = 5 experiments, 332 cells), T47D (n = 4 experiments, 187 cells), MDA-MB-321 (n = 4 experiments, 192 cells), prostate (rose, RWPE-1 (n = 5 experiments, 124 cells), PC-3 (n = 4 experiments, 153 cells) and skin (green, melanocytes, n = 3 experiments, 651 cells), 1205Lu (n = 5 experiments, 618 cells). Points= means of independent experiments. (*F*) TEM micrographs of NEs in melanocyte (*Upper*) or 1205Lu (*Lower*) cells. (*G*) Quantification NE spacing from TEM images, n = 400 measurements in 8 melanocytes, 866 measurements in 11 1205Lu cells. In (*A*–*C*) bar = 10 µm, in (*E*) Bar = 1 µm. Statistical significance tested with Student’s *t* test Mann–Whitney (*D*), Student’s *t* test Kolmogorov–Smirnov (*F*). Error bars: (*D* and *F*) mean; (*D*) min and max, (*E*) = 25th to 75th percentile.

We first sought to determine if NE gene expression was commonly altered in cancer, and if this related to NE fragility and nuclear mechanics. We examined the expression of 249 NE genes from the Human Protein Atlas in 6,860 clinical samples representing 10 cancer subtypes in The Cancer Genome Atlas (TCGA) PanCancer repository (*SI Appendix*, Fig. S1*A* and Table S1). Dysregulation occurred across cancer types, consistent with previous studies (24). To test if these transcriptional changes related to NE fragility, we used a polydimethylsiloxane (PDMS) cellular confinement device mimicking in vivo tissue confinement (25) in cells expressing two different biosensors for NE fragility. To assess changes in NE permeability that allowed release of soluble nucleoplasmic proteins into the cytoplasm, we expressed mCherry fused to a nuclear localization signal (mCherry-NLS). This probe remains soluble in the nucleoplasm, but leaks into the cytosol if the NE loses compartmentalization due to small rapidly repaired breaches (26), nuclear pore dilation or stretching (27, 28), or activation of membrane pore-forming proteins (29) (Fig. 1 *A*–*C*). To assess large-scale NE rupture extensive enough to allow chromatin spillage into the cytoplasm and DNA exposure to damage by cytosolic DNAses (30), we expressed GFP-tagged cyclic GMP-AMP synthase (GFP-cGas). This probe is soluble in the cytosol and binds rapidly to dsDNA when chromatin breaches the NE (6, 8, 10), forming stable fluorescent foci at NE rupture sites (8). We subjected representative benign melanocytes and malignant melanoma cells (1205Lu) to three different confinement heights (2.4 µm, 3 µm, and 5 µm) (*SI Appendix*, Fig. S1 *B* and *C*). We found that cell nuclei were insensitive to 5 µm confinement but showed both NE permeability and rupture at 2.4 µm confinement, while at 3 µm confinement, malignant cells exhibited significantly more NE rupture than benign, despite no difference in unconfined nuclear height (*SI Appendix*, Fig. S1*D*). Assaying cell lines representing benign and malignant breast and prostate tissues using a 3 µm confinement height (Movie S1) confirmed that malignant cells had significantly more fragile NEs, compared to their benign counterparts in other tissues (Fig. 1 *A*–*E*). Thus, dysregulation of NE-associated genes and increased NE fragility under confinement are common intrinsic properties of malignant cells.

### NE and Membrane-Related Genes Are Transcriptionally Upregulated during Melanoma Disease Progression.

To determine how transcriptional alterations in NE genes mediate changes in NE fragility in cancer, we focused on melanoma, as its skin accessibility has made tissue samples and expression profile datasets from staged patient biopsies widely available. Cell morphometric analysis of 100 patient biopsies revealed significantly lower nuclear solidity in benign nevi compared to stage 1-2 or metastatic tumors, indicating abnormal nuclear morphology is an early indicator of melanoma progression (*SI Appendix*, Fig. S1 *E* and *F*). Transmission electron microscopy indicated the INM-ONM spacing in benign melanocytes was uniform ~45 nm in width, while melanoma cells displayed inconsistent membrane spacing ranging from 4-177 nm (Fig. 1 *F* and *G*). Thus, alterations in nuclear morphology and NE architecture correlate with melanoma malignant transformation.

**Fig. 2.**
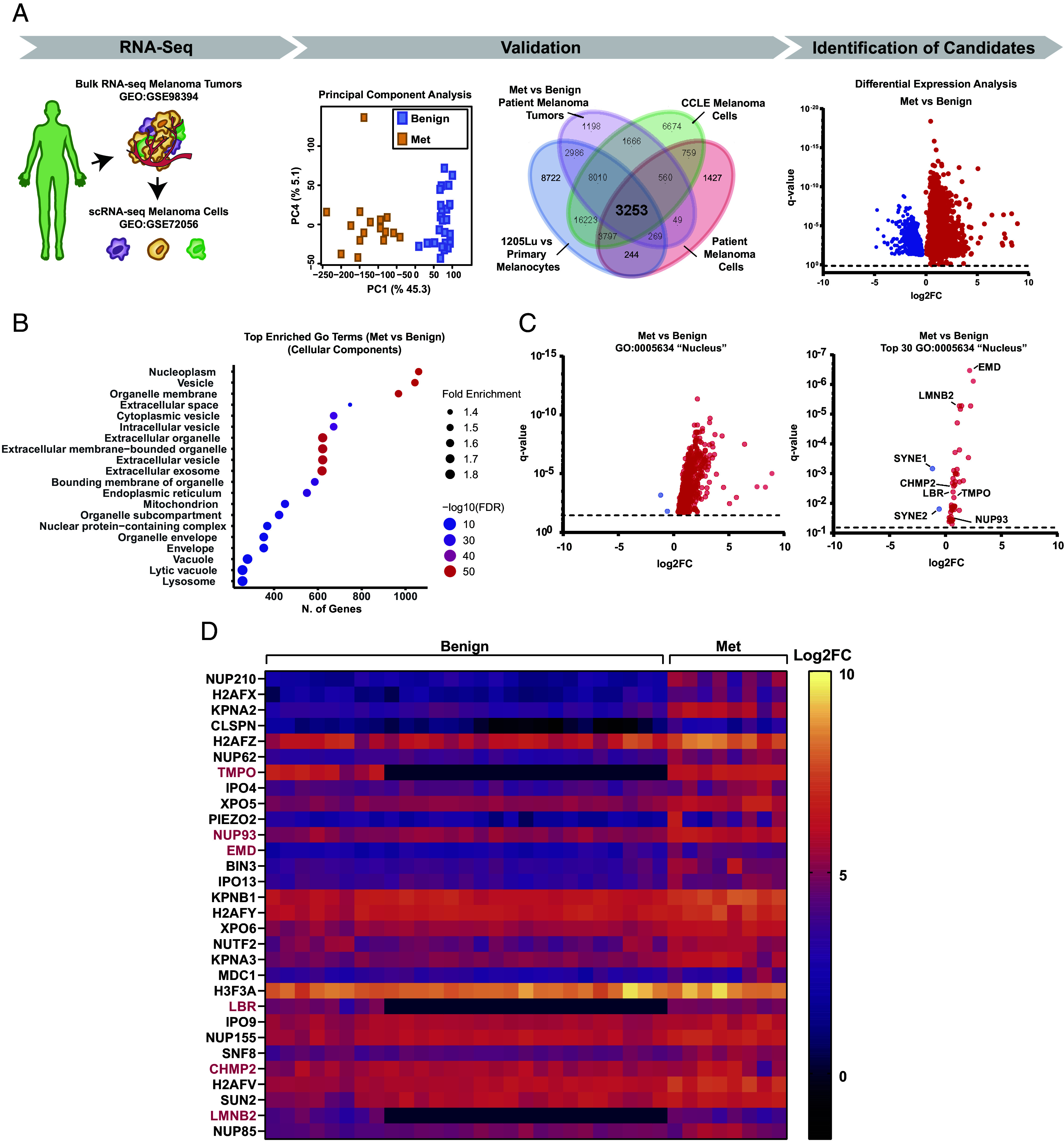
Transcriptional alterations in NE genes correlate with melanoma disease progression. (*A*) Bioinformatic data analysis pipeline comparing RNA-seq of benign nevi to stage III and IV tumors (metastatic melanoma). Principal component analysis (*Middle Left*) between benign nevi and metastatic melanoma. Cross-referencing to find genes in common with genes differentially expressed between human melanocytes and 1205Lu cells and 27 melanoma cell lines from Broad cancer cell line encyclopedia (CCLE, *Middle Right*, gene numbers in each subset shown), resulting in 3,253 genes common to all datasets. Volcano plot DEA of the 3,253 gene subset, comparing benign nevi and metastatic melanoma in patient biopsies (GEO: GSE98394) (red = significantly upregulated, blue = significantly downregulated) (far right). (*B*) Gene ontology (GO) enriched cellular component terms in the common gene subset (3,253). (*C*) Volcano plot DEA of genes grouped from the functional annotation by GO analysis (utilizing DAVID) in the cellular component term “Nucleus” (GO:0005634, *Left*), and of the 30-most enriched genes in the Nucleus ranked by false discovery rate (FDR) score (*Right*). Red = significantly upregulated, blue = significantly downregulated. (*D*) Heat map from RNA-seq data from individual patient biopsies for the 30-most enriched Nucleus genes, pink text: genes of interest chosen for siRNA-based screen.

We next utilized a bioinformatic approach to analyze BRAF^V600E^ melanomas from all clinical stages and melanoma cell lines, to identify common changes in transcriptional state occurring during disease progression. Principal component analysis (PCA) of an RNA-seq transcriptomic dataset (GEO:GSE98394) from bulk tumor biopsies identified a highly significant separation of components between benign nevi and metastatic melanoma, indicating melanoma progression from early to aggressive is a clear transcriptional evolution (Fig. 2*A* and *SI Appendix*, Fig. S2 *A* and *B*). To exclude transcripts from nonmalignant cells (i.e. fibroblasts, immune cells) in bulk tumors, we utilized single-cell (sc) RNA-seq data from FACS-sorted melanoma cells in staged tumors (GEO:GSE72056) (Fig. 2*A*). We then performed a meta-analysis on transcripts that were differentially expressed between benign nevi and metastatic melanoma in both bulk tumors and FACS-sorted melanoma cells. We cross-referenced this patient-derived subset against genes differentially expressed between immortalized melanocytes and 1205Lu melanoma cells, as well as from the transcriptomes of 27 melanoma cell lines from the Broad cancer cell line encyclopedia (CCLE) (Fig. 2*A*). Genes common to all these datasets were subjected to differential expression analysis (Fig. 2*A*), thus identifying 3,253 candidate genes that differed significantly in expression between BRAF^V600E^ benign and metastatic melanomas. Applying gene ontology enrichment and functional annotation clustering analysis revealed the enrichment of nucleoplasmic and membrane-associated genes (Fig. 2*B*). Interestingly, nearly all the nuclear-associated genes were upregulated in metastatic melanoma compared to benign, with the exception of the LINC complex components nesprin-1 and nesprin-2, that were significantly downregulated (Fig. 2 *C* and *D*). Together, these results show that nuclear and NE-related abnormalities associated with aggressive melanoma correlate with upregulation of nuclear genes in patient tumor biopsies.

### LBR Upregulation Is Necessary and Sufficient for Promoting NE Fragility and Nuclear Deformability.

**Fig. 3.**
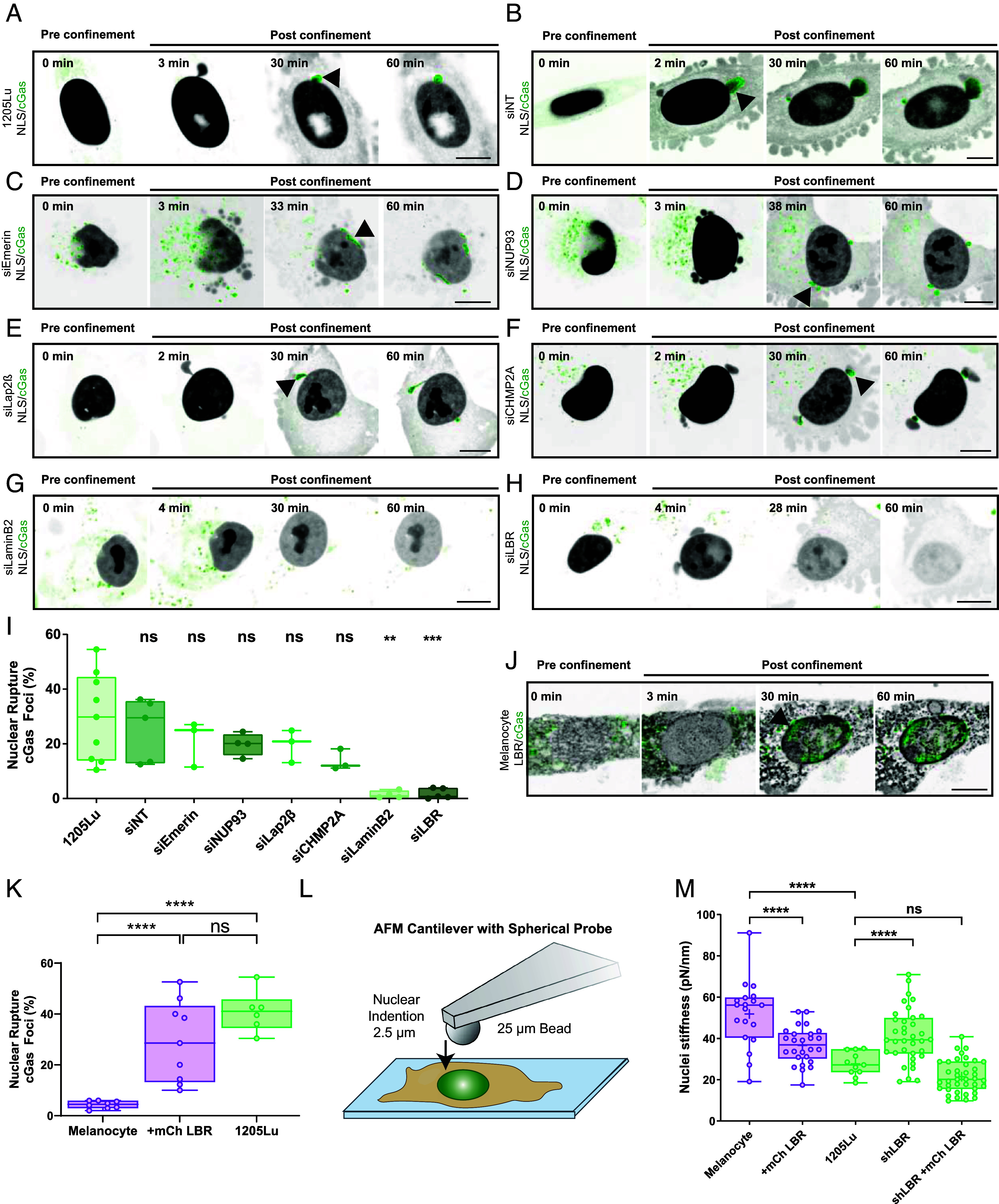
siRNA-based screen identifies LBR as necessary and sufficient for promoting NE fragility and nuclear deformability under confinement. (*A*–*H*) Confocal image series of 1205Lu cells with mCherry-NLS (inverted grayscale) and GFP-cGas (green) alone (*A*) or together with either nontargeting siRNA (*B*, siNT) or siRNAs targeting emerin (*C*, siEmerin), NUP93 (*D*, siNUP93), Lap2β (*E*, siLap2β), CHMP2A (*F*, siCHMP2A), laminB2 (*G*, siLaminB2), or LBR (*H*, siLBR), before (pre confinement), and after (post confinement) confinement to 3 µm. Arrows highlight a perinuclear GFP-cGas foci, imaging time noted. (*I*) Quantification of NE rupture in 1205Lu cells with or without (1205Lu, n = 9 experiments, 2,473 cells) cotransfection with nontargeting siRNA (siNT, n = 5 experiments, 421 cells) or siRNA targeting emerin (siEmerin, n = 3 experiments, 261 cells), NUP93 (siNUP93, n = 4 experiments, 521 cells), Lap2β (siLap2β, n = 3 experiments, 195 cells), CHMP2A (siCHMP2A, n = 3 experiments, 204 cells), LaminB2 (siLaminB2, n = 4 experiments, 607 cells), or LBR (siLBR, n = 5 experiments, 1,627 cells). (*J*) Image series of a melanocyte with mCherry-LBR (inverted gray scale) and GFP-cGas (green) before (pre confinement) and after (post confinement) confinement to 3 µm. Arrow highlights GFP-cGas foci, imaging time noted. (*K*) Quantification of NE rupture in 1205Lu cells (green, n = 6 experiments, 947 cells) or melanocytes (purple) with (+mCherry LBR, n = 8 experiments, 876 cells) or without mCherry LBR (n = 9 experiments, 1,845 cells). (*L*) AFM experimental set up. (*M*) Quantification of bulk nuclear stiffness from melanocytes (purple) (n = 3 experiments, 19 cells) or 1205Lu cells (green) (n = 3 experiments, 11 cells) with LBR shRNA (LBR KD, n = 4 experiments, 36 cells) and/or mCherry-LBR (Melanocytes: + mCh LBR n = 3 experiments, 25 cells; 1205Lu: LBR KD +mCh LBR n = 5 experiments, 37 cells). In (*A*–*H* and *J*) bar = 10 µm. In (*I* and *K*) points = means of individual experiments, (*M*) points = individual cells. In (*I* and *K*) statistical significance was tested with Student’s *t* test Mann–Whitney, in (*M*) Student’s *t* test using Welch’s correction, bars = means; boxes = 25th to 75th percentile, error bars = min and max.

We next sought to establish if transcriptional modulation of nuclear-associated genes alters NE fragility during cancer progression. We selected a high priority subset from the top 30 upregulated NE genes in our bioinformatic analysis (Fig. 2 *C* and *D*) and reduced their expression by siRNA, including: Lamina-associated polypeptide 2b (Lap2β) and emerin, both of which bind to lamins and regulate gene expression and organization (31); Lamin B2, an intermediate filament protein of the lamina that confers NE structural support and regulates genome organization (32); Nup93, a scaffold protein in the nuclear pore complex (33); CHMP2A, an interacting partner of endosomal sorting complex required for transport (ESCRT-III) protein that facilitates NE repair after mitosis (34); and lamin B receptor (LBR), an integral INM protein consisting of TUDOR and RS domains tethering it to lamin B and heterochromatin (35, 36) and a C-terminal 8-spanning transmembrane domain that contributes to cholesterol biosynthesis via its sterol reductase activity, mutations in which are associated with human disease (37). Cells were cotransfected with nontargeting (NT) or siRNAs targeting the gene of interest together with mCherry-NLS or GFP-cGas, subjected to confinement at 3 µm, and the effects of knockdown (KD) on markers of NE fragility quantified (Fig. 3 *A*–*H* and Movie S2). This revealed that reduction of Lap2β, CHMP2A, or laminB2 significantly decreased NE permeability (*SI Appendix*, Fig S3*A*), while KD of either laminB2 or LBR nearly abolished NE rupture (Fig. 3*I*). These results indicate that transcriptional upregulation of NE components during melanoma progression promotes NE fragility in confinement and can drive either NE permeabilization or large-scale NE rupture.

To determine the mechanism by which NE gene upregulation causes NE rupture, we focused on LBR. Validation of our bioinformatics result by western blot confirmed that LBR was 30% more abundant in 1205Lu cells compared to immortalized melanocytes, and either pooled siRNA oligonucleotides or lentiviral-induced shRNAs targeting the 3’UTR of LBR (LBR-KD) reduced its level in 1205Lu cells to that similar to melanocytes (*SI Appendix*, Fig. S3 *B* and *C*). Importantly, reexpressed mCherry-LBR in LBR-KD cells localized to the NE and ER (*SI Appendix*, Fig. S3*D*), and restored confinement-induced cGas foci frequency to that of control 1205Lu cells (*SI Appendix*, Fig. S3*E*). To determine if increasing LBR alone was sufficient to promote NE fragility and deformability in nonmalignant cells, we subjected immortalized melanocytes expressing GFP-cGas, together with or without mCherry LBR, to cell confinement. We found that moderate overexpression of mCherry LBR increased the frequency of NE rupture in melanocytes by more than 3.5-fold, raising it to the level observed in 1205Lu cells (Fig. 3 *J* and *K*, *SI Appendix*, Fig. S3 *F* and *G*, and Movie S3). Thus, upregulation of LBR in melanoma cells specifically promotes NE fragility in confinement, and its overexpression is sufficient to cause NE rupture in benign melanocytes.

We then asked whether upregulation of LBR in melanoma affects nuclear mechanical properties. We utilized simultaneous AFM and confocal microscopies to quantify bulk nuclear stiffness and visualize nuclear shape. 1205Lu cells cotransfected with GFP-H2B to mark the nucleus and NT or LBR-KD siRNAs, either alone or together with mCherry-LBR, were indented 2.5 µm with a bead-affixed AFM cantilever directly on top of their nuclei (Fig. 3*L*). This resulted in repeatable and reversible bimodal force–displacement curves as the cantilever penetrated deeper into the cell, with the first mode representing the contribution of the plasma membrane and cortical cytoskeleton and second mode representing the bulk nuclear component, as it was sensitive to KD of laminA (38) and less sensitive to KD of laminB1 (39) (Fig. 3*L* and *SI Appendix*, Fig. S3 *H*–*J*). AFM analysis showed that nuclei of immortalized melanocytes were significantly stiffer than those of 1205Lu melanoma cells, and LBR-KD in 1205Lu caused bulk nuclear stiffness to increase to the level in melanocytes, while reexpression of mCherry-LBR in LBR-KD cells or overexpression of mCherry-LBR in melanocytes both decreased stiffness to the level in 1205Lu (Fig. 3*M*). Thus, increased LBR expression is necessary and sufficient to cause NE rupture in confinement and generate a softer, more deformable nucleus.

### LBR Upregulation in Metastatic Melanoma Causes INM Fragility.

We then sought to determine the cellular mechanism by which excess LBR causes NE rupture. To examine the role of LBR in maintaining substructures of NE architecture, we compared immunostaining of the lamin nucleoskeleton (lamins A/C, B1 and B2), the INM (Lap2β, LBR), the LINC complex (Sun2), nuclear pores (Nup358, Nup214, and Nup153), and condensed heterochromatin (H3K9me2, H3K9me3, H4K20me3) in WT versus LBR-KD 1205Lu cells. Confocal imaging and quantification of staining intensity at the NE showed that LBR-KD caused no significant difference in the levels of Sun2, nuclear pores, lamins B1 and B2, or condensed heterochromatin. LBR depletion did cause minor but statistically insignificant reductions in lamin A/C and Lap2β levels in the NE (*SI Appendix*, Fig. S4*A*). Thus, proteins involved in maintaining NE architecture are not regulated by LBR level.

**Fig. 4.**
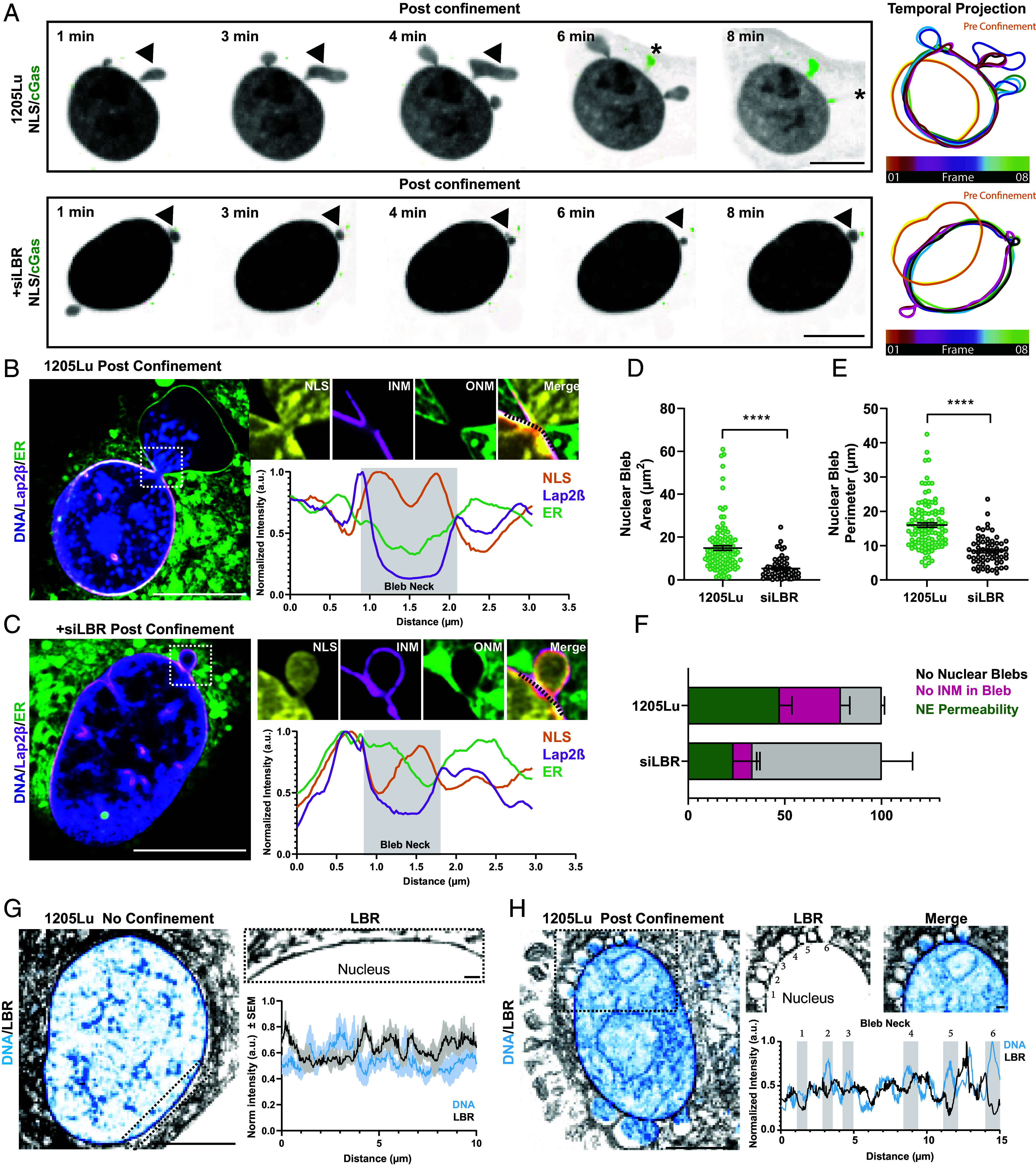
LBR promotes nuclear rupture through INM instability. (*A*, *Left*) Confocal image series of 1205Lu cells cotransfected with mCherry-NLS (inverted gray scale) and GFP-cGas (green) together with (*Bottom* row) or without (*Top* row) siRNAs targeting LBR (+siLBR) after (post confinement) confinement to 3 µm. Arrows: NE blebs, asterisks: burst NE blebs, time after initiation of imaging noted. (*Right*) Color-encoded temporal projection of the outline of the nucleus over the first 10 min of confinement, orange: preconfinement. (*B* and *C*) Superresolution confocal images during confinement to 3 µm of living 1205Lu cells transfected with mCherry-NLS (yellow) and GFP-Lap2β (magenta) and stained with blue-white ER-Tracker (green) and SiR DNA (blue); with (*C*) or without (*B*) transfection with siRNAs targeting LBR (+siLBR). Zooms of white dashed boxed regions (above, *Right*), intensity linescans of black dotted lines (below, *Right*), gray box: position of the NE bleb neck (Bleb Neck). (*D* and *E*) Quantification of NE bleb area (*D*) and perimeter (*E*) from cells treated as in (*B* and *C*). 1205Lu: green, n = 3 experiments, 116 cells; siLBR: gray, n = 3 experiments, 137 cells. Data points represent individual cells. (*F*) Quantification of fraction of cells exhibiting no NE blebbing (gray), no INM within an ONM bleb (pink) or NE permeability (green) from time-lapse movies of cells treated as in (*B* and *C*) 1205Lu: n = 4 experiments, 16 cells; siLBR: n = 4 experiments, 16 cells. (*G* and *H*) Superresolution confocal images of 1205Lu cells transfected with mCherry-LBR (inverted gray scale) and stained with Hoechst (cyan), with (*H*) or without (*G*) confinement to 3 µm. Zooms of black dotted boxed regions (above, *Right*), intensity linescans (below, *Right*), positions of six NE bleb necks (Bleb Necks) numbered (1–6) and gray boxes. In (*G*) line traces n = 5 cells. In (*A*–*C*, *G*, and *H*) bars = 10 µm. In (*D* and *E*) statistical significance was tested with Student’s *t* test Mann–Whitney, in (*F*) Student’s *t* test using Welch’s correction. In (*D* and *E*) bars = means, error bars = min and max, in (*F*) error bars = SEM.

To determine how chromatin breached the lamina, INM and OMN, we first performed immunostaining of laminA/C in 1205Lu cells coexpressing mCherry-NLS and GFP-cGas, and with DNA labeled with DAPI. In confined cells positive for nuclear rupture as indicated by mCherry-NLS in the cytoplasm and perinuclear cGas foci, we found that laminA/C staining was absent from the site where DNA protruded from the nucleus into the cytoplasm and cGas foci formed, indicating breach of the lamina (*SI Appendix*, Fig. S4*B*). We next probed the organization and dynamics of the INM and ONM during confinement-induced NE rupture in living cells by expressing our nuclear fragility biosensors together with GFP-Lap2β to mark the INM (40, 41), and vital staining of DNA and the ER/ONM membrane with SiR-DNA and a dye that binds K^+^ channels in the ER, respectively. Analysis of time-lapse images revealed that while both WT and LBR-KD 1205Lu cells generated nuclear blebs during confinement, those in WT cells were larger and contained chromatin, compared to LBR-KD cells which exhibited smaller nuclear blebs with little-to-no DNA staining. Time-lapse superresolution imaging revealed that the NLS-containing blebs formed at the surface of 1205Lu cell nuclei were enclosed in ER/ONM-positive membrane that was contiguous with the NE, suggesting it was an extension of the ONM (Fig. 4 *A*–*C*). These ONM blebs grew and filled with mCherry-NLS and DNA over time until the ONM membrane ruptured, releasing mCherry-NLS and chromatin into the cytosol (Fig. 4 *A*–*C* and Movies S1 and S2). Comparison of the INM marker GFP-Lap2β and the ER/ONM marker showed a small discontinuity in the GFP-Lap2β that formed locally concomitant with the establishment of the ER/ONM-positive bleb (Fig. 4 *B* and *C*, *SI Appendix*, Fig. S4*C*, and Movie S4), which remained largely devoid of INM marker as it expanded and subsequently burst. While local Lap2β exclusion at the bleb neck could be due to a membrane diffusion barrier or other mechanisms, the likely cause is rupture of the INM. In contrast, nuclear blebs in LBR-KD cells were encased by both GFP-Lap2β and ER/ONM-positive membranes (Fig. 4 *B*–*F* and Movie S4), and often resolved over time with both INM and ONM markers remaining contiguous (Fig. 4 *A*–*F*). These results suggest upregulation of LBR in confined melanoma cells causes ONM blebbing that is potentially initiated by local breakage of the lamina and INM, and confinement-induced pressure subsequently drives extrusion of chromatin into the bleb and eventual rupture of the ONM.

We next sought to determine how LBR organization could contribute to INM rupture. We analyzed endogenous LBR localization in unconfined 1205Lu melanoma cells by immunofluorescence and observed the presence of LBR clusters interspersed with LBR-deficient regions in the INM (*SI Appendix*, Fig. S4 *D* and *E*), similar to previous reports (42). Analysis of LBR staining intensity variance along the NE periphery indicated reduction of LBR by siRNA resulted in a significant decrease in LBR variance, suggesting reduced clustering, compared to 1205Lu cells (*SI Appendix*, Fig. S4 *E* and *F*). Superresolution and line-scan analysis of DNA and mCherry-LBR in the NE of unconfined 1205Lu cells showed that intensity peaks of mCherry-LBR in the NE anticorrelated with peaks in peripheral DNA, indicating LBR clusters formed in heterochromatin-depleted regions of the nuclear periphery (Fig. 4*G*). Line scan analysis of confined cells exhibiting NE blebs showed that LBR appeared enriched at the bleb necks where chromatin spillage occurred (Fig. 4*H*). Thus, upregulation of LBR in melanoma promotes its clustering at chromatin-depleted regions of the INM, where during confinement, local breakage of the INM and pressure-induced blebbing rupture the ONM to ultimately spill chromatin into the cytoplasm.

### LBR Sterol Reductase Activity Promotes NE Fragility.

**Fig. 5.**
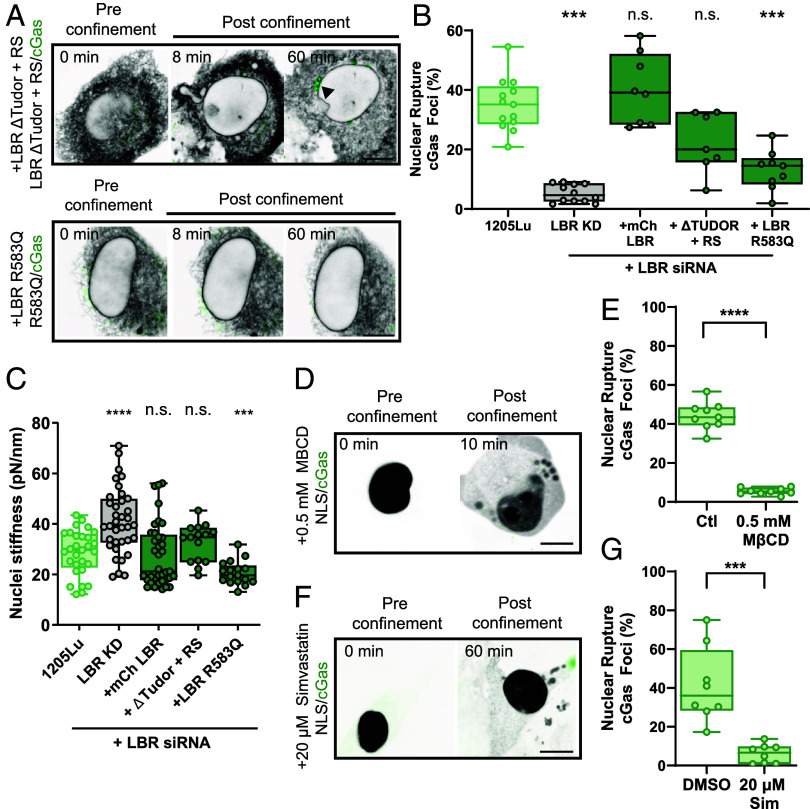
Disease-associated mutation in LBR reveals sterol reductase activity promotes NE fragility and cholesterol production. (*A*) Confocal image series before (pre confinement)and after (post confinement)confinement to 3 µm of 1205Lu cells cotransfected with GFP-cGas (green) and siRNA targeting the 3’UTR of LBR together with either mCherry-LBRΔTudor+RS or mCherry LBR 583Q (inverted grayscale). Arrow highlights cGas focus. (*B*) Quantification of fraction of confined cells exhibiting NE rupture in 1205Lu cells expressing GFP-cGas alone (light green, 1205Lu, n = 6 experiments, 746 cells) or together with LBR siRNA (gray, LBR KD, n = 4 experiments, 3,283 cells) and either mCherry LBR (dark green, +mCherry LBR n = 3 experiments, 1,342 cells), mCherry-LBRΔTudor+RS (dark green, n = 3 experiments, 610 cells), or mCherry-LBR 583Q (dark green, n = 3 experiments, 1,142 cells). (*C*) Quantification of bulk nuclear stiffness in 1205Lu cells (light green, n = 5 experiments, 30 cells) expressing LBR siRNA (gray, LBR KD, n = 4 experiments, 36 cells) and either mCherry LBR (dark green, n = 5 experiments, 37 cells), mCherry-LBRΔTudor+RS (dark green, n = 3 experiments, 15 cells), or mCherry-LBR 583Q (dark green, n = 3 experiments, 17 cells). Data points represent individual cells (*D*) Confocal image series of 1205Lu cells treated with 0.5 mM MβCD and expressing mCherry-NLS (inverted grayscale) and GFP-cGas (green), before and after confinement to 3 µm. (*E*) Quantification of the fraction of confined cells exhibiting NE rupture in 1205Lu cells in media (Ctl, n = 3 experiments, 738 cells) or treated with 0.5 mM MβCD (n = 3 experiments, 1,329 cells) and expressing GFP-cGas. (*F*) Confocal image series of 1205Lu cells treated with 20 µM simvastatin and expressing mCherry-NLS (inverted grayscale) and GFP-cGas (green), before and after confinement to 3 µm. (*G*) Quantification of the fraction of confined cells exhibiting NE rupture in 1205Lu cells expressing GFP-cGas and treated with vehicle (+DMSO, n = 3 experiments, 443 cells) or 20 µM simvastatin (n = 3 experiments, 559 cells). In (*A*, *D*, and *F*) bar = 10 µm. In (*B*, *E*, and *G*) significance was tested with Student’s *t* test Mann–Whitney, in (*C*) Student’s *t* test using Welch’s correction, in (*C*) error bars = min and max. In (*B*, *E*, and *G*) significance was tested with Student’s *t* test with Kolmogorov–Smirnov correction. Bars = means, error bars = SD, boxes= 25th to 75th percentile.

To define the molecular mechanism mediating LBR’s promotion of NE fragility and nuclear deformability, we utilized a mutant add-back approach in an LBR-KD background together with our assays for nuclear rupture and mechanics. We generated an mCherry-tagged truncation mutant that lacked the N-terminal lamin- and direct chromatin-binding domains (mCherry-LBR-ΔTudor+RS) and a Greenberg’s skeletal dysplasia-associated point mutant (mCherry-LBR-R583Q) which abolishes the sterol reductase activity (43, 44) (Fig. 5*A* and *SI Appendix*, Figs. S3*F* and S5*A*). Analysis of NE rupture in confinement and bulk nuclear deformability by AFM of LBR-KD 1205Lu cells expressing mCherry-LBR-ΔTudor+RS revealed that this mutant rescued the effects of LBR-KD on cGas foci formation and nuclear deformability, similar to LBR-KD cells expressing mCherry-LBR (Fig. 5 *B* and *C* and Movie S5). Similarly, AFM analysis of LBR-KD cells expressing mCherry-LBR-R583Q showed that this mutant rescued the decrease in nuclear deformability caused by LBR depletion, inducing nuclear softening to a level even greater than that of either 1205Lu cells or LBR-KD cells rescued with mCherry-LBR (Fig. 5*C*). In surprising contrast, mCherry-LBR-R583Q expression was unable to rescue the decrease in NE rupture under confinement caused by knockdown of LBR (Fig. 5 *B* and *C* and Movie S5). These results show that lamin and direct chromatin tethering by LBR are dispensable for LBR’s promotion of NE fragility and nuclear deformability, while LBR’s C-14 sterol reductase activity promotes NE fragility but not nuclear deformability, indicating NE fragility and nuclear stiffness are governed by mechanistically distinct functions of LBR.

To determine how LBR’s sterol reductase activity promotes NE fragility under confinement, we examined the contribution of LBR to cholesterol biosynthesis in melanoma cells. We first verified that neither perturbation of LBR nor depletion of cholesterol from the media affected expression of LBR’s ER-localized homologue, TM7SF2, both of which act post-lanosterol in the cholesterol biosynthetic pathway reducing F-MAS, to produce T-MAS (*SI Appendix*, Fig. S5*B*). We then confirmed that LBR-KD caused disruption of cholesterol biosynthesis by GC-MS analysis of sterols in lysates of cells cultured in lipid-depleted media, which showed that LBR-KD cells exhibited a small peak representing buildup of cholesta-8,14-dien-3β-ol, similar to that seen in cells from patients with Greenberg’s skeletal dysplasia and milder nonlethal skeletal dysplasias resulting from pathogenic variants in LBR (45), indicating depletion of LBR causes a deficiency in its enzymatic activity (*SI Appendix*, Fig. S5*D*). However, neither this intermediate nor other cholesterol biosynthetic intermediates accumulated in WT 1205Lu cells, indicating buildup of precursor sterols is not the cause of LBR-mediated NE fragility.

We next sought to determine if LBR upregulation in metastatic melanoma altered total cellular cholesterol levels, and if cholesterol itself modulated NE fragility. Fluorometric analysis of lysates of 1205Lu or LBR-KD cells alone or reexpressing either mCherry-LBR or mCherry-LBR-R583Q cultured in lipid-free media showed that LBR-KD significantly reduced total cellular cholesterol, and rescue of LBR-KD with mCherry-LBR restored cholesterol almost to control levels, while rescue with the sterol reductase-deficient mutant did not (*SI Appendix*, Fig. S5*C*). To determine if acute reduction of cholesterol was sufficient to reduce NE rupture in confined melanoma cells, we treated 1205Lu cells expressing our NE fragility biosensors with methyl-β-cyclodextrin (MβCD) which rapidly removes cholesterol from cellular membranes (46). Subjecting MβCD-treated cells to confinement showed that cholesterol depletion caused nuclei to appear much more deformable and fluid-like and significantly decreased the occurrence of GFP-cGas foci compared to control cells, without affecting NE permeability, similar to effects of LBR-KD (Fig. 5 *D* and *E*, *SI Appendix*, Fig. S5 *F* and *H*, and Movie S6). Depletion of cholesterol with the clinically relevant drug simvastatin, which targets HMG CoA reductase upstream of C-14 sterol reductase in the cholesterol biosynthetic pathway, also caused confined cells’ nuclei to appear deformable and decreased occurrence of GFP-cGas foci, similar to effects of acute MβCD (Fig. 5 *F* and *G*, *SI Appendix*, Fig. S5 *E*, *G*, and *I*, and Movie S7). Thus, upregulation of LBR during melanoma disease progression increases cellular cholesterol to drive NE fragility under confinement, and reduction of cholesterol levels is sufficient to abrogate this effect.

### LBR Promotes Invasion and NE Rupture during Confined Migration in Tumor Organoids.

**Fig. 6.**
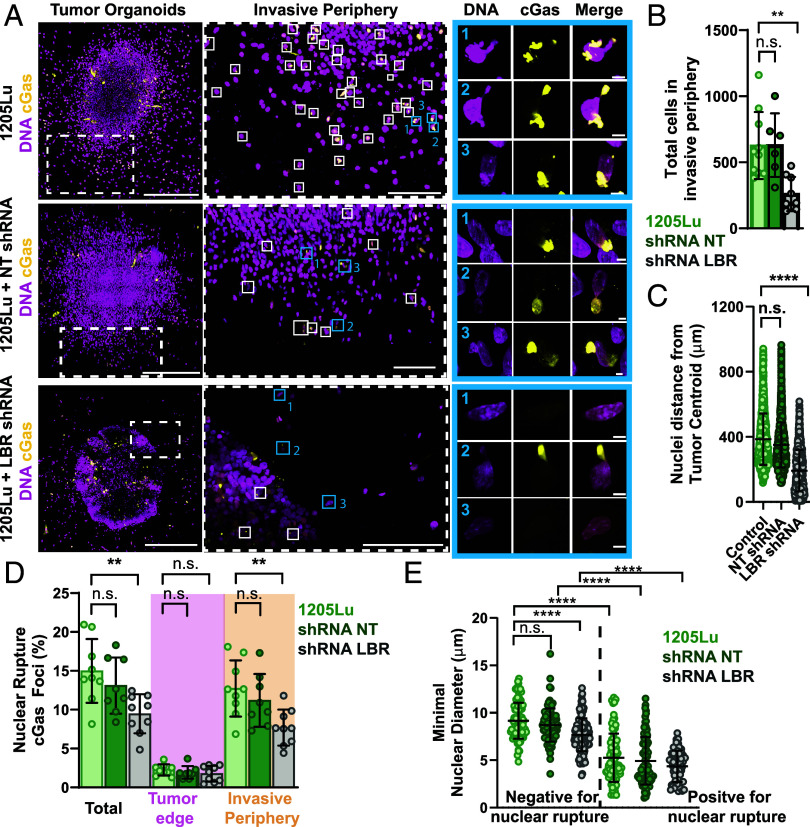
LBR promotes nuclear rupture in cells migrating out of tumor organoids embedded in collagen. (*A*) 3D projections of z-series of confocal images of tumor organoids grown for 7 d in a collagen ECM from 1205Lu cells stably expressing GFP-cGAS (yellow) and labeled with Hoechst (magenta) without (1205Lu, *Top* row) or with stable expression of either nontargeting shRNA (1205Lu+NT shRNA, *Middle* row) or shRNA targeting LBR (1205Lu+LBR shRNA, *Bottom* row). White dotted boxed regions representing invasive periphery from left are zoomed (center), white boxed regions in center indicate cells positive for cGas foci, numbered blue boxed regions from center are zoomed (*Right*). (*B*–*E*) Quantification of (*B*) total number of cells in the invasive periphery; (*C*) Distance of nuclei from the organoid center; (*D*) Percent of nuclei exhibiting rupture in the total organoid (Total, *Left*), at the tumor edge (dusky rose, center), and in the invasive periphery (gold, *Right*); (*E*) Smallest nuclear diameter in cells with (*Right*, Negative for nuclear rupture) and without (*Left*, Positive for nuclear rupture) cGas foci; for organoids grown from 1205Lu cells stably expressing GFP-cGAS and mScarlet-NLS with or without (1205Lu, bright green) stable expression of nontargeting shRNA (shRNA NT, dark green) or shRNA targeting LBR (shRNA LBR, gray) for 7 d in a collagen ECM. Data points represent individual cells (*C* and *E*) or independent experiments each consisting of pooled cell data (*B* and *D*). In (*A*) bar = 500 µm (far left), 150 µm (*Middle*), and 5 µm (far right). In (*B* and *D*) statistical significance was tested with Student’s *t* test Mann–Whitney, (*C*) Student’s *t* test using Welch’s correction, in (*E*) significance was tested with ANOVA. Bars = means with SD, in (*B*–*E*), (*D*) error bars = min and max boxes = 25th to 75th percentile.

We next examined the role of LBR in NE fragility and melanoma invasion in tumor organoids in vitro. 1205Lu cell lines stably expressing GFP-cGas and mScarlet-NLS along with inducible LBR-KD or NT-shRNAs (*SI Appendix*, Fig. S6*A*) were embedded in 2.5 mg/mL 3D collagen matrices to provide a confining ECM (47). After allowing cells to proliferate and invade the ECM for 1 wk, analysis of 3D confocal imaging showed that compared to WT 1205Lu and NT controls, LBR depletion significantly decreased both the number of cells that detached from the tumor and invaded the ECM and the distance they traveled from the organoid center, although overall organoid volume was unaffected (Fig. 6 *A*–*C* and *SI Appendix*, Fig. S6*E*). While high cell density precluded accurate measurement of NE rupture in organoid cores, quantification of perinuclear GFP-cGas foci in a 250 µm-thick 3D shell around the organoid edge revealed a low base level of NE rupture in this region present in all conditions (Fig. 6*D*). However, outside the organoid edge, many WT 1205Lu or NT that had invaded the ECM displayed GFP-cGas foci, while invasive LBR-KD cells showed significantly less cells with NE rupture (Fig. 6 *A*, *D*, and *E*, *SI Appendix*, Fig. S6*D*, and Movies S8 and S9). This difference in rupture was despite the fact that organoids exhibited similar collagen meshwork density and architecture in all conditions (*SI Appendix*, Fig. S6 *B* and *C*). Analysis of the minimal diameter of nuclei as a proxy for nuclear deformation showed that those nuclei associated with GFP-cGas foci were significantly skinnier than those without, independent of condition (Fig. 6*E*), suggesting that rupture was specific to nuclei subjected to confinement by the ECM or other cells. Furthermore, in LBR-KD cells, we never observed DNA blebbing from the nuclear periphery without a cGas focus present, indicating LBR-KD does not affect cGas recognition of DNA (Fig. 6*A*). These results indicate that transcriptionally upregulated LBR promotes increased cell invasion and NE rupture in invasive cells in melanoma tumor-like organoids.

### LBR Upregulation Promotes Invasion in Melanoma Tumors In Vivo and Is Associated with Shorter Disease-free Survival in Patients.

**Fig. 7.**
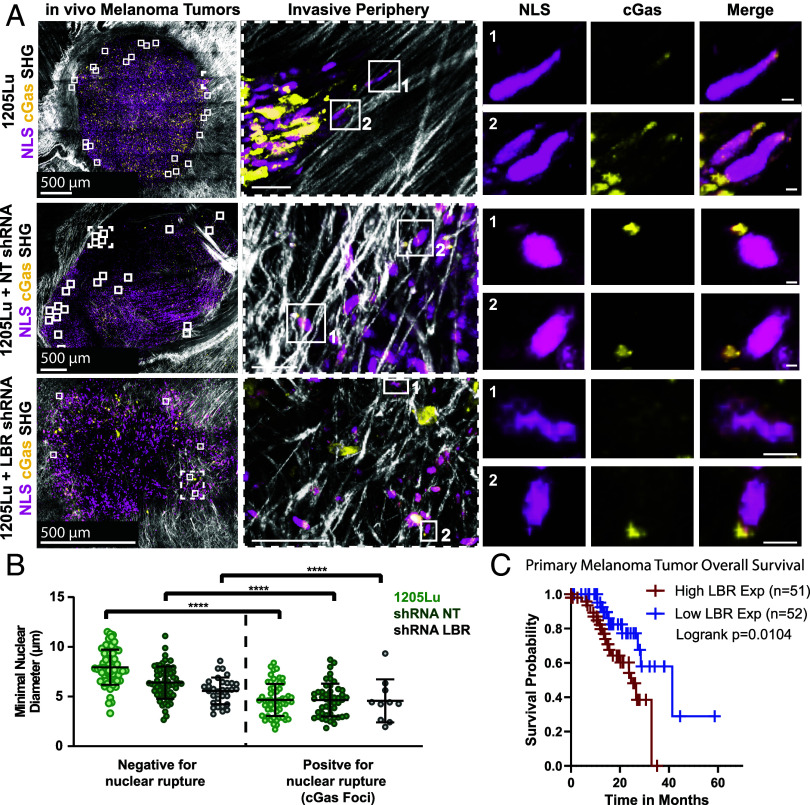
LBR promotes nuclear rupture during invasion in vivo. (*A*) Z projections of two-photon confocal images of 1205Lu tumors expressing GFP-cGAS (green) and mScarlet-NLS (magenta) with (LBR shRNA) or without (1205Lu) shRNAs targeting LBR. Boxes (*Left*) cells positive for perinuclear cGas foci. Boxes from invasive periphery are zoomed (*Left Center*) numbers indicate nuclei at (*Right*). (*B*) Quantification of tumor cell nuclear diameter with (*Right*, Positive for nuclear rupture) and without (*Left*, Negative for nuclear rupture) cGas foci, for 1205Lu tumors expressing GFP-cGAS and mScarlet-NLS with or without (1205Lu, bright green) expression of nontargeting shRNA (shRNA NT, dark green) or shRNA targeting LBR (shRNA LBR, gray). (*C*) Kaplan–Meier survival probability for SKCM patients from TCGA, color coded by LBR expression level in Primary melanoma tumors. Data points represent individual cells (*B* or *C*) individual tumor samples. In (*A*) bar = 500 µm (note different zoom for *Bottom* row), 50 µm (*Middle*), and 5 µm (far right). In (*B*) significance was tested with ANOVA (*C*) log-rank test. Bars = means with SD.

We next asked whether upregulation of LBR promotes NE fragility and rupture in melanoma tumors in vivo, and if this correlates with disease severity. We injected 1205Lu cells expressing LBR- or NT-shRNA together with the mScarlet-NLS and GFP-cGas biosensors intradermally into Foxn1^nu^ mice and allowed tumors to grow and invade for two months (Fig. 7*A*). Harvesting tumors for high-resolution 2-photon imaging ex vivo revealed substantial ECM rearrangement and melanoma cell migration in all experimental conditions, as indicated by the presence of thick bands of collagen peripheral to the tumor edge and cells appearing to use both single and collective migratory modes to exit the primary tumor (Fig. 7*A*). Analysis of cell number and tumor volume in 3D reconstructions of whole tumors revealed a significant decrease in cell density within LBR-KD tumors compared to NT or WT tumors, but no effect on overall tumor volume (*SI Appendix*, Fig. S7 *A*–*C*). While resolution limitations and background fluorescence deep in tissue precluded quantification of all cells with perinuclear GFP-cGas foci, we did note that nuclei-associated GFP-cGas foci occurred predominantly at the tumor periphery, specifically in cells metastasizing through dense collagen, and nuclear rupture appeared more frequent in control tumors compared to LBR-KD (Fig. 7 *A* and *B*, *SI Appendix*, Fig. S7*D*, and Movies S10 and S11). Quantification of minimal nuclear radius revealed that nuclei with cGas foci were significantly skinnier than those without foci, independent of condition (Fig. 7*B*), implying NE rupture occurred in cells experiencing confinement in vivo. These results show that transcriptionally upregulated LBR in melanoma tumors in mouse skin promotes increased cell invasion and NE rupture in vivo.

We next asked if LBR expression level correlated with patient survival. Analysis of our bulk tumor transcriptomics dataset showed that LBR expression significantly increased early in melanoma, during progression from benign nevi to stage 1 and 2 tumors, and continued to be elevated in stage 3 and 4 tumors and in metastases (*SI Appendix*, Fig. S7*E*). We then queried patient melanoma survival data from staged melanoma tumors using the SKCM dataset from TCGA. Although LBR level was not associated with significantly decreased disease-free survival in samples from all tumor stages or from secondary tumors, (*SI Appendix*, Fig. S7*F*), we found that LBR upregulation in primary melanoma was predictive of significantly decreased survival (Fig. 7*C*). Collectively, these data demonstrate that excess LBR in BRAF^V600E^ melanoma promotes tumor growth and invasion, and drives NE fragility and nuclear deformability specifically during invasive confined metastatic migration, and if upregulated early in melanoma development is linked to worse patient prognosis.

## Discussion

We performed a systematic analysis of reccurring transcriptional changes and cell confinement assays in benign and malignant cancer cells, finding that upregulation of NE genes and increased NE fragility are intrinsic to cancer progression from benign to metastatic in three cancer subtypes. Focusing on melanoma using a bioinformatics approach, we found that upregulation of nucleoplasm, vesicle, and organelle membrane genes marked melanoma progression from benign nevi to metastatic. A confinement-based siRNA screen identified upregulation of LBR as necessary and sufficient for promoting increased NE fragility and deformability in melanoma. Live cell imaging showed that NE rupture occurs through a three step cellular mechanism. First, LBR upregulation drives its clustering in the INM where the INM ruptures. The pressure of confinement then squeezes nucleoplasm and chromatin into a large ONM bleb, which eventually ruptures, resulting in spillage of nucleoplasm and exposure of chromatin to the cytosol, thus increasing its likelihood for DNA damage. Molecular mechanistic studies revealed LBR’s sterol reductase activity contributes to excess cellular cholesterol, and this activity is responsible for promoting INM fragility under confinement. Our analysis of physiological models of melanoma invasion both in vitro and in vivo show that LBR expression promotes increased invasion through confined ECMs, where cells are subjected to confinement and nuclear deformation, causing NE rupture. Thus, LBR upregulation during melanoma disease progression causes increased NE fragility through its enzymatic role in cholesterol biosynthesis, promoting increased metastatic invasion.

How LBR clusters promote INM fragility is not clear. LBR consists of an N-terminal tail that binds lamins and chromatin both directly and indirectly (48), and a C-terminal transmembrane domains that acts as a sterol reductase in the cholesterol biosynthetic pathway (49). We found that LBR formed clusters in heterochromatin-depleted regions at the nuclear periphery (Fig. 4), which is surprising considering its documented binding activities. However, as both the lamina and heterochromatin are known to provide structural support and stiffness to the nucleus (16, 39), this suggests that sites of LBR clusters that were enriched at NE bleb necks may correspond to chromatin-depleted weak points where INM rupture could occur. In line with this, we found that the lamin and chromatin binding activities were not required for LBR’s promotion of NE fragility (16, 39). Importantly, we found that a point mutant that abolishes LBR’s sterol reductase activity and pharmacological depletion of cholesterol inhibited NE rupture. We suggest that LBR-mediated biosynthesis of cholesterol could create cholesterol-rich regions in the NE, causing local defects between cholesterol-poor regions which may be prone to rupture when exposed to mechanical stress. Thus, LBR clusters may cause NE rupture by locally excluding chromatin and enriching cholesterol, weakening the INM membrane.

The finding that LBR-mediated cholesterol production causes NE fragility is intriguing in the context of cancer, as high cholesterol has been associated with tumor development (50) and immune cell invasion in melanoma (51). Furthermore, epidemiological studies have shown that long-term statin use to decrease serum cholesterol is associated with decreased cancer progression and severity in many cancer subtypes (52), including melanoma (53). Additionally increased cholesterol synthesis driven by upregulated LBR could serve as a metabolic enhancer, increasing tumor cells’ ability to proliferate and cope with nutrient-deprived conditions (44). Together, our findings suggest that LBR could be a prognostic indicator in early melanoma disease progression, and could serve as a drug target to prevent metastatic dissemination of melanoma, thereby improving prognosis for patient survival.

## Materials and Methods

1205Lu cells (a kind gift from Glenn Merlino, Laboratory of Cancer Biology and Genetics, CCR, NCI, NIH, Bethesda, MD 20892) were cultured in Tu 2% media and supplemented with 2% fetal bovine serum (FBS; Atlanta Biologicals S11150) 2.5 ng/mL insulin (Sigma), and 1.68 mM CaCl_2_. Immortalized human melanocytes (Glenn Merlino, NCI) were cultured in dermal cell basal media (ATCC, PCS-200-030), supplemented with melanocyte growth kit (ATCC, PCS-200-041). MCF10A (ATCC, CRL-10317), PC-3 (ATCC, CRL-1435), HEK293FT (ATCC, CRL-3216), and T47D (NIH, NCI-60) cells were all cultured in DMEM/F12+GlutaMax (Gibco) with 10% fetal bovine serum (Atlanta Biologicals), and 1% penicillin and streptomycin (ThermoFisher, 10378016). RWPE-1 cells were cultured in keratinocyte serum-free media supplemented with 25 mg of bovine pituitary extract and 2.5 µg epidermal growth factor (ThermoFisher, 17005042). To reproducibly confine cells during high-resolution live-cell imaging, confinement was performed using the 1-well Dynamic Cell Confiner System (4Dcell) per the manufacturer’s directions. Animal protocols and care were performed as directed by the National Heart Lung and Blood Institute and approved by the Institutional Animal Care and Use Committees guidelines. Detailed information relating to the methodology and reagents used for cell and organoid culture, cDNA and lentiviral expression vectors, ex vivo tumor models, bioinformatics analysis (RNA-seq and TCGA data acquisition and analysis), cellular confinement assays, immunostaining, microscopy, image analysis, measurement of cellular cholesterol, western blots and all statistical analysis are provided in the *SI Appendix, Materials and Methods*.

## Supplementary Material

Appendix 01 (PDF)

Dataset S01 (PDF)

Movie S1. Confocal image series of benign and malignant cells before and after confinement 3μm. Representative cell lines for skin (immortalized human melanocytes, 1205Lu), breast (MCF10A or T47D) and prostate (RWPE-1, PC-3) transfected with mCherry-NLS (inverted grayscale) and GFP-cGas (green). Arrows highlight GFP-cGas foci indicating nuclear rupture. Time interval between images 60 s. Scale bar, 10μm.

Movie S2. Confocal image series of 1205Lu cells transfected with mCherry-NLS (inverted grayscale) and GFP-cGas (green) with and without treatment with the following siRNA’s to specifically deplete NE proteins; 1205Lu WT (top, left), nt siRNA (top, center left), emerin (top, center right), NUP93 (top right), Lap2b (bottom left), CHMP2A (bottom center left), laminB2 (bottom center right) or LBR (bottom right), before and after confinement to 3mm. Arrows highlight GFP-cGas foci indicating nuclear rupture. Time interval between images 120 s. Scale bars, 10μm.

Movie S3. Confocal image series of immortalized human melanocytes transfected with mCherry-NLS or mCherry-LBR (inverted grayscale) and GFP-cGas (green) before and after confinement to 3μm. Arrows highlight GFP-cGas foci indicating nuclear rupture. Time interval between images 120 s. Scale bar, 10μm.

Movie S4. Superresolution Z-series of a 1205Lu transfected with EGFP- Lap2b (magenta), and vital dyes blue-white ER tracker (green) and siR-DNA (blue) after confinement to 3mm, highlighting differential rupture of the INM and ONM. Scale bar as shown in movie. Superresolution Z-series of a 1205Lu transfected with siRNA depleting LBR, EGFP- Lap2b (magenta), and vital dyes blue-white ER tracker (green) and siR-DNA (blue) after confinement to 3mm, highlighting differential rupture of the INM and ONM. Scale bar as shown in movie.

Movie S5. Confocal image series of 1205Lu cells transfected with GFP-cGas (green) and treated and siRNA targeting the 3’UTR of LBR (+LBR siRNA) together with either mCherry-LBR (left, inverted grayscale), mCherry-LBRDTudor+RS (left center, inverted grayscale), mCherry LBR 583Q (right center, inverted grayscale) or mCherry LBR 1402DT (right end, inverted grayscale), before and after confinement to 3 μm. Arrows highlight GFP-cGas foci indicating nuclear rupture. Time interval between images 120 s. Scale bar, 10 μm.

Movie S6. Confocal image series of 1205Lu cells transfected with mCherry-NLS (inverted grayscale) and GFP-cGas (green) treated with either vehicle control (2% Tu Media) or 0.5 mM MβCD, before and after confinement to 3 μm. Arrow indicates cGas focus, indicating a cell positive for nuclear rupture. Time interval between images 120 s. Scale bar, 10 μm.

Movie S7. Confocal image series of 1205Lu cells transfected with mCherry-NLS (inverted grayscale) and GFP-cGas (green) treated with either vehicle control (DMSO) or 20 μM simvastatin, before and after confinement to 3mm. Arrow indicates cGas focus, indicating a cell positive for nuclear rupture. Time interval between images 120 s. Scale bar, 10 μm.

Movie S8. 3D reconstruction of a 17.5μM-thick confocal Z-series of fixed tumor organoids grown from 1205Lu cells stably expressing GFP-cGas (yellow) and mScarlet-NLS (magenta) in a collagen ECM. Scale bar as shown in movie.

Movie S9. 3D reconstruction of a 36μM-thick confocal Z-series of fixed tumor organoids grown from 1205Lu cells stably expressing GFP-cGas (yellow), mScarlet-NLS (magenta) and shRNA targeting LBR, in a collagen ECM. Scale bar as shown in movie.

Movie S10. 3D reconstruction of a 223μM-thick two-photon confocal Z-series of living ex-vivo tumors grown from 1205Lu cells stably expressing GFP-cGAS (yellow) and mScarlet-NLS (magenta) in mouse dermis. Scale bar as shown in movie. Gamma correction was applied to SHG channel (greyscale) to allow for better visualization of dim collagen fibers.

Movie S11. 3D reconstruction of a 145μM-thick two-photon confocal Z-series of living ex-vivo tumors grown from 1205Lu cells stably expressing GFP-cGAS (yellow), mScarlet-NLS (magenta) and shRNA targeting LBR in mouse dermis. Scale bar as shown in movie. Gamma correction was applied to SHG channel (greyscale) to allow for better visualization of dim collagen fibers.

## Data Availability

Previously published data were used for this work (GEO: GSE98394 and GSE72056). Other data are included in the article and/or supporting information.
