# Supplementary material for: Lamin B receptor upregulation in metastatic melanoma causes nuclear envelope fragility in confined migration during cancer invasion
Source: Proc Natl Acad Sci U S A. 2026 Feb 18;123(8):e2513031123. doi: 10.1073/pnas.2513031123 (PMC12933115; doi:10.1073/pnas.2513031123)
Supplement: Supplementary file 1 — Appendix 01 (PDF) [file pnas.2513031123.sapp.pdf]

PNAS

Supporting Information for

Lamin B Receptor Upregulation in Metastatic Melanoma Causes Nuclear Envelope Fragility in Confined Migration During Cancer Invasion

Michelle A. Baird*, Cayla E. Jewett, Daniela A. Malide, Lisa Kratz, Alexander X. Cartagena-Rivera, Robert S. Fischer, and Clare M. Waterman*

* Michelle A. Baird, Clare M. Waterman

Emails: michelle.baird@nih.gov, watermancm@nhlbi.nih.gov

This PDF file includes:

- Supporting text
- Figures S1 to S7
- Legends for Movies S1 to S11
- SI References 1-23

Other supporting materials for this manuscript include the following:

- Movies S1 to S11
- Table S1

Materials and Methods

Cell and Organoid Culture

1205Lu cells (a kind gift from Dr. Glenn Merlino, NCI) were cultured in Tu 2% media (80% MCDB153, 20% L-15) supplemented with 2% fetal bovine serum (FBS; Atlanta Biologicals S11150) 2.5 ng/ml insulin (Sigma), and 1.68mM $CaCl_2$. Immortalized human melanocytes (Dr. Glenn Merlino, NCI) were cultured in dermal cell basal media (ATCC, PCS-200-030), supplemented with melanocyte growth kit (ATCC, PCS-200-041). MCF10A (ATCC, CRL-10317), PC-3 (ATCC, CRL-1435), HEK293FT (ATCC, CRL-3216) and T47D (NIH, NCI-60) cells were all cultured in DMEM/F12 + GlutaMax (Gibco) with 10% fetal bovine serum (Atlanta Biologicals), and 1% penicillin and streptomycin (ThermoFisher, 10378016). RWPE-1 cells were cultured in keratinocyte serum-free media supplemented with 25 mg of bovine pituitary extract and 2.5 µg epidermal growth factor (ThermoFisher, 17005042). All cells were maintained at 37°C at 5% CO_2 for <15 passages. Transient expression of cDNAs or siRNAs was performed using the Amaxa nucleofector kit R (Lonza) and Amaxa Nucleofector II (Lonza) programs X-001 (1205Lu), U-024 (melanocytes), or the Amaxa Nucleofector kit V (Lonza) and Amaxa Nucleofector II (Lonza) programs T-020 (MCF10A), T-013(PC-3, RWPE-1), Q-001 (Hek293FT), X-005 (T47D) and 1.5 µg of DNA for $2x10^6$ cells.

To generate melanoma organoids, 2000 cells were seeded in 200 µl of media in low adherence U-bottom 96 well dishes (Nunclon Sphera, Thermo Fisher) and allowed to proliferate for 7 days. To embed organoids in ECM, 3 thin layers of polymerized collagen were used to control Z height and placement of organoids in a cell culture dish. Briefly, 35 mm glass bottom dishes (FluoroDish, WPI) were plasma cleaned then coated with a thin layer of 2.5 mg/ml Type 1 collagen (Corning) which was allowed to polymerize at 37°C for 1 hour. Following polymerization, organoids were gently aspirated from 96 well dishes using a pipette and individually placed in the center of the 35mm dish and encased with an additional layer of 2.5 mg/ml of collagen that was allowed to polymerize at 37°C for 1 hour. Following the second layer, a final layer of collagen was applied to ensure full coverage of the organoids and attachment of the collagen to the dish sidewalls. It was treated similarly to the other layers. Upon final polymerization, 1 ml of culture media was applied to each dish and organoids were maintained for 1 week to allow for proliferation and cell invasion into the ECM.

CDNA expression vectors and lentiviral expression

The following cDNAs were used for DNA transfections or subcloning (1, 2**)**: mCherry NLS, mCherry-N1, and mEmerald-H2B were kind gifts of the late Mike Davidson (Florida State University, Tallahassee, FL); GFP-cGas (a kind gift from Dr. Hawa Racine Thiam, Stanford); GFP-Lap2b (Addgene 62044); EGFP-LBR (Addgene 128150); pTrip-CMV-GFP-Flag-cGas (Addgene 86675); pLBS.CAG-NLS-mScarlet (Addgene 129336). To generate mCherry-LBR, LBR cDNA was PCR amplified to generate NheI and EcoRI restriction sites and an 18 amino acid linker using primers (Fw-CGTTCCGCTAGCGCC ACCATGCCAAGTAGGAAATTTGCCGATGGTGAAGTGGTAAGAGGTC) (Rev-CCA GACGAATTCCGCTGTAGATGTATGGAAATATACGGTAGGGCACACGCTGACAGT AC). To generate mCherry-LBR, LBR cDNA was PCR amplified to generate NheI and EcoRI restriction sites and an 18 amino acid linker using primers (Fw- CGTTCCGCT AGCGCCACCATGCCAAGTAGGAAATTTGCCGATGGTGAAGTGGTAAGAGGTC) (Rev- CCAGACGAATTCCGCTGTAGATGTATGGAAATATACGGTAGGGCACACGCT GACAGTAC). To generate mCherry-LBR-ΔTudor+RS, the reverse primer above was

reused and the following forward primers was used (CGTTCCGCTAGCGCCACCATG GGTCGACCACCTAAAAGTGCCCGCCGATCT). The obtained inserts and mCherry-N1 were digested with restriction enzymes (NheI and EcoRI, New England Biolabs) then ligated with T4 DNA ligase (ThermoFisher, EL001) and treated with calf intestinal alkaline phosphatase (CIAP, ThermoFisher, 18009019) and gel purified (QIAquick Gel Extraction Kit; Qiagen) and transformed into DH5α competent cells (ThermoFisher, 18258012). All colonies were then miniprepped (Qiagen) and confirmed by sequencing, then large scale purified (Qiagen) for experimental use. To generate mCherry-LBR-R583Q, site directed mutagenesis was used (QuikChange II XL Site Directed Mutagenesis Kit, Agilent, 200521) as directed. The primers used to introduce the appropriate mutations into mCherry-LBR-N-18 were (5'-ctcgtcacgagcttcttggtggacaagcaacat-3'), (5'-atgttgcttgtccaccaagaagctcgtgacgag-3'). All colonies were then miniprepped (Qiagen) and confirmed by sequencing, then large scale purified (Qiagen) for experimental use. For knockdown (KD) of candidate nuclear envelope (NE) proteins for screening for their effects on nuclear fragility in confinement, ON-TARGETplus SMARTpool siRNAs (Dharmacon) were used for human LBR (J-021505-05), emerin (J-011025-06) Nup93(J-020767-09), Lap2β (J-027195-09), CHMP2A (L-020247-01), laminB2 (L-005290), laminA (L-004978), and non-targeting (NT) pool (D-001810-10). For siRNA screen, protein knockdown was verified by western blot for LBR and NT pool. For LBR rescue experiments, a custom 3'UTR duplex was utilized (5'GUAAAGGAGUGCUGUCUUAUU 3') (Dharmacon). For all siRNA experiments, cells were transiently transfected with 1 µM of siRNA for 48 hours prior to experimental use.

Generation of a custom LBR shRNA lentivirus plasmid was done using the above 3'UTR duplex RNA sequence to design a shRNA ligated into the pLKO-Tet-On lentivirus AgeI-EcoRI sites for expression under the control of the H1/TO promoter (3). As a control, inducible NT expressing lentivirus was used (Dharmacon). 1205Lu cells stably expressing LBR shRNA (Dharmacon) and NT shRNA (Dharmacon) were generated using the second generation lentiviral packaging plasmid psPax2 (Addgene 12260) and pMD2.G (Addgene 12259). Lentiviral particles were generated by first transfecting 1 million HEK293FT with Lipofectamine 2000 (Thermo Fisher) according to manufacturer's protocols using 2.5 µg plasmid DNA, 1 µg pMD2.G, and 2.5 µg psPax2 and collecting the virus-containing supernatant at 48 hours. Supernatant was then filtered through a 0.45 µm filter (Sigma), then immediately placed on target 1205Lu cells that had been seeded 24 hours before transduction at a 1:1 ratio of viral supernatant to 2% Tu media and allowed to incubate for 48 hours, upon which cells were passaged into media containing a final concentration of 2.0 µg/ml puromycin for selection. KD was induced in cell culture using 100 nM doxycycline (Sigma) for 48 hours prior to experiments.

Generation of stable cell lines

1205Lu cells stably expressing pTrip-CMV-GFP-Flag-cGas (Addgene 86675) and pLBS.CAG-NLS-mScarlet (Addgene 129336) were generated using the second generation lentiviral packaging plasmid psPax2 (Addgene 12260) and pMD2.G (Addgene 12259) as described above. Following 2.0 µg/ml puromycin selection, cells were then expanded for 2 weeks before being sorted by flow cytometry for GFP- and mScarlet-positive cells.

***Ex vivo* tumor models**

Tumors were generated by intradermal injection of 5000 human metastatic melanoma cells (1205Lu) in anaesthetized Crl:NU(NCr)-Foxn1nu (athymic nude mice; Charles River, Rockville MD). Cells stably expressing GFP-cGas and mScarlet-NLS were injected in a 1:1 mixture of Matrigel and minimal essential medium (Gibco) and imaged once tumors were between 4-5 mm in size. For tumor imaging, animals were euthanized and dorsal skin was removed with at least 10 mm of margin around the tumor. The superficial fat layer was removed and tumors were treated with Hoechst 33342 (1:1000) for 10 min at RT, then whole-mounted tumors *in situ* were imaged in dermal cell basal media supplemented with melanocyte growth kit. Animal protocols and care were performed as directed by the National Heart Lung and Blood Institute and approved by the Institutional Animal Care and Use Committees guidelines.

Drug Treatments

The following pharmacological inhibitors were used:
Simvastatin: (20 µM, dissolved in DMSO; Sigma); Methyl-beta-cyclodextrin (MβCD): (0.5mM dissolved in water; Sigma).

RNA-Sequencing

RNA from 1205Lu cells and immortalized human melanocytes were isolated and purified using RNeasy Micro Kit (Qiagen) according to manufacturer's instruction and concentration was quantified using a NanoDrop Spectrophotometer (ThermoScientific). Sequencing libraries were constructed from 100– 500 ng of total RNA using Illumina's TruSeq Stranded Total RNA Kit with Ribo-Zero according to manufacturer's instructions. The fragment size of RNAseq libraries was verified using a 2100 Bioanalyzer (Agilent) and the concentrations were determined using a Qubit instrument (LifeTech). The libraries were first run on an Illumina Miseqkit for QC purposes. After QC analysis, the libraries were loaded onto an Illumina HiSeq 3000 for 2 x 75-bp paired end read sequencing. The fastq files were generated using the bcl2fastq software for further analysis.

Bioinformatics

The Cancer Genome Atlas (TCGA) Data Acquisition

Unbiased identification of potential nuclear membrane genes of interest was obtained using the uniform manifold approximation and projection (4) (UMAP) subcellular distribution analysis from The Human Protein Atlas (5). Querying the 'Nuclear Membrane' category resulted in the identification of 249 nuclear membrane-associated genes. Next, The Cancer Genome Atlas (TCGA) Pan-Cancer dataset (6) was accessed through the University of California, Santa Cruz (UCSC) Xena Browser (http://xena.ucsc.edu/), and was queried for 10 cancer subtypes; Bladder Urothelial Carcinoma (BLCA), Breast invasive carcinoma (BRCA), Colon adenocarcinoma (COAD), Head and Neck squamous cell carcinoma (HNSC), Kidney renal papillary cell carcinoma (KIRP), Liver hepatocellular carcinoma (LIHC), Lung adenocarcinoma (LUAD), Lung squamous cell carcinoma (LUSC), Prostate adenocarcinoma (PRAD) and Stomach adenocarcinoma (STAD). The subset of 249 NE genes identified from the Human Protein Atlas was surveyed in the Xena Browser then exported for subsequent data visualization with Prism.

Identification of NE genes of interest from patient melanoma samples

RNA-seq data from patient bulk melanoma tumours was downloaded from GEO (GSE98394) and categorized based on provided clinical staging information into benign nevi (27 samples), stage 1 and 2 (35 samples), and stage 3 and 4 (32 samples, referred to as “metastatic melanoma”) (7). Clinical staging information was based on both the Tumor, Nodes, Metastasis classification of malignant tumors (TNM) and American Joint Committee on Cancer (AJCC). Melanoma single cell RNA-seq (scRNA-seq) data was downloaded from GEO (GSE72056) and data from malignant cells were isolated (8). Melanoma cell line RNA-seq data was downloaded from the Cancer Cell Line Encyclopedia (CCLE) and date from malignant melanoma samples were isolated (9). All RNA-seq data was subjected to quality control analysis using FastQC (v0.11.8) (10) . This was followed by alignment to the human genome (GRCh38/hg38) using spliced transcripts aligned to a reference (STAR) (11). Gene expression was quantified using featureCounts (v2.0.0) (12). RNAseq sample quality was determined using ESTIMATE (v 1.0.13) R package. Tumor purity estimates and sex were used as the covariates for differential expression analysis, performed using limma-voom (13). Genes with significantly differential expression with q value < 0.05 were subjected to pathway enrichment analysis using clusterProfiler (v3.14.3) (14). All RNA-seq data was filtered to remove genes with an expression count < 5 in 90% of the samples, and FDR< 0.05, to remove low-expressing and non-significant genes. Integration of data sets was performed in the following order: bulk tumor (GEO: GSE98394), single cell melanoma samples (GEO: GSE72056), and Broad CCLE melanoma cell lines. Genes were retained if they were present in all datasets, and consistently up- or down-regulated between all samples, forming “Group 1”. Following this, differential expression analysis (DEA) was performed as previously described (15) between 1205Lu and immortalized human melanocyte (IM) cells, to identify transcriptomic changes occurring in 1205Lu as compared to IM. The DEA was then integrated with the previously described Group 1 using the same criteria as listed above. The final gene set was then named “Group 2” and used for analysis. To provide a comprehensive view of genes enriched from the gene set from Group 2, the gene list was input into two separate algorithms using gene ontology (GO) cellular components annotation and enrichment terms.

The first algorithm is based on the Ensembl gene annotation and enrichment (16) and the ShinyGo 0.82 (17) graphical visualization tools, and provided annotation categories featuring enrichment from the input gene set. For this analysis the false discovery rate (FDR) cut off was set at 0.05 and the resulting gene enrichment categories were sorted by FDR as presented in Fig. 2B. The second analysis focused on grouping genes enriched in “Group 2” into functional annotation clustering, identifying gene clusters based on their functional similarity. For this analysis we utilized the Database for Annotation, Visualization, and Integrated Discovery (DAVID) (18), (classification stringency- medium, threshold 0.5, and Benjamini statistical analysis), which resulted in the identification of 332 clusters of enrichment with the top 5 as follows (Cytoplasm; enrichment score 135.71, GO:0005737 ; Intracellular anatomical structure; enrichment score 87.7, GO:0005622; Cytosol; enrichment score 63.89, GO:0005829; Nucleus; enrichment score 42.09, GO:0005634; and Membrane, GO:0016020; enrichment score 31.39). To determine if expression of genes that were identified in the Nucleus GO:0005634 functional cluster from Group 2 changed during melanoma progression, we analysed their expression levels in benign nevi *vs* stage 3-4 tumors from the bulk melanoma tumor dataset (GEO: GSE98394), resulting in the volcano plot in Fig. 2C. The top 30 nuclear genes present within GO:0005634 by FDR are highlighted in the clinical

sample data from GEO:GSE98394 in Fig 2C right, and Fig. 2D. Additional ranking of genes by q-value and fold change, and targeted segregation of nuclear genes that have a known role in nuclear envelope mechanics resulted in our final list of high priority gene candidates for the siRNA screen.

Confinement assays

To reproducibly confine cells during high-resolution live-cell imaging, confinement was performed using the 1-well Dynamic Cell Confiner System (4Dcell) per manufacturer's directions and as previously described (19). Briefly, the system consists of a cobalt autonomous vacuum pump attached to a manufactured PDMS suction cup fitted with a glass coverslip containing PDMS micropillars of 2.5, 3, or 5 μm height which determine the spacing between the top of the coverslip and the cell culture dish. To perform confinement experiments, cells were transiently transfected and plated for 24-48 hours (depending on experimental conditions) on 35mm glass bottom dishes (FluoroDish, WPI) coated with 10 μg/ml fibronectin. Prior to imaging, the PDMS suction cup and coverslip were briefly sonicated in 70% ethanol, followed by a 5-minute sonication in PBS (Invitrogen), then placed in cell culture media for 1 hour at room temp to equilibrate the PDMS. To confine the cells, the 35mm glass bottom dish was washed briefly with pre-warmed PBS, then 1 ml of fresh warmed cell culture media was added. The sample was then placed on the microscope and the PDMS suction cup was attached by a low pressure vacuum seal that was sufficient for attachment of the PDMS perimeter base to the glass dish, but insufficient for cellular confinement. Confinement was initiated over 1 minute using a ramp of 10 mbar per second utilizing the custom software (4Dcell) to control the vacuum pump. After cells were confined, images were acquired from multiple (10-40) XY positions to ensure full unbiased coverage of the confined area, images were acquired every 1-2 minutes over 1 hour for time-lapse analysis.

Immunofluorescence

Immunostaining of adherent cells

Cellular immunofluorescence for NE proteins was performed using 1205Lu cells plated on cleaned coverslips (#1.5, 22 mm x 22 mm, Corning) for 24-72 hours depending on experimental conditions. Samples were then fixed for 20 min at 37°C with 4% paraformaldehyde (PFA; Electron Microscopy Science,15710) in cytoskeleton buffer (CB; 10 mM MES, 3 mM $MgCl_2$, 138 mM KCl, 2 mM EGTA), permeabilized with 0.5% Triton X-100 in CB at room temperature (RT) for 5 mins, and quenched with 10 mM glycine in CB at RT. Cells were washed 2 x 5 mins then 2 x 10 mins with Tris Buffered Saline (TBS; 20 mM Tris, pH 7.6, 137 mM $NaCl_2$) before blocking for 1 hour at RT with blocking solution (2% BSA IgG free and protease free (Sigma-Aldrich, A3059); 0.1 % Tween 20 (Sigma-Aldrich, P1379) in TBS). Cells were incubated for 1.5 hours at RT with the following primary antibodies: LBR (1:200, Atlas Antibodies HPA062236), LaminB1 (1:500, Abcam ab16048), LaminB2 (1:500, Abcam ab8983), Sun2 (1:500, Abcam ab87036), H4K20me2 (1:500, Abcam ab9052), H3K9me2 (1:500, Active Motif AB 2793199), Lap2 (1:1000, BD Transduction Laboratories 611000), Mab414 (1:1000, Abcam ab24609), Lamin A/C (1:500, Abcam ab108595) or HP1 (1:500, Abcam ab109028), diluted in blocking solution then washed in TBS 3 x10 mins before being incubated with fluorophore-conjugated secondary antibodies (1:500), Alexa Fluor 488 Donkey anti-rabbit (711-545-5152), Alexa Fluor 594 Donkey anti-rabbit (711-585-152), Alexa Fluor 488 Donkey anti-mouse (715-545-150) and Alexa Fluor 594 Donkey anti-mouse (715-585-150) and Dapi (1μg/mL; Sigma, 268298) diluted in blocking solution, for

1 hour at RT. Cells were washed with blocking solution with TBS (2 x 10 mins, each). Coverslips were mounted on glass slides in mounting media (Dako; Pathology Products, S3023).

Immunostaining of confined cells

Immunostaining for laminA in confined 1205Lu cells was performed in cells plated on 35mm glass bottom dishes (FluoroDish, WPI) and confined to 3µm. Samples were then fixed during confinement for 60 min at 37°C with 4% paraformaldehyde (PFA; Electron Microscopy Science,15710) in CB to allow fixative to penetrate the PDMS cell confiner. Following fixation, the cell confiner was removed and dishes were washed 2 x 5 min with TBS to remove cell debris then treated as described above.

Immunostaining of organoids

Tumor organoids embedded in collagen in 35mm glass-bottom dishes were fixed for 60 min at 37°C with 4% paraformaldehyde in CB then permeabilized with 0.5% of Triton X-100 in CB at 4°C overnight. Organoids were then washed 4 x 15 mins with TBS before blocking at 4°C overnight. Organoids were then incubated at 4°C overnight with Dapi (1µg/mL; Sigma, 268298) diluted in blocking solution. Organoids were washed with blocking solution then with TBS (2 x 10 mins, each).

Immunostaining of tissue microarrays

Tissue microarrays (TMAs) of melanoma progression (ME1004f, US Biomax) were used for visualization and quantification of nuclear morphology alterations during cancer progression. Paraffin was removed through a series of xylene and ethanol dilution rinses: 100% xylene, 3 x 5 min, 100% ethanol, 2 x 5 min, 95% ethanol, 1 x 5 min, 70% ethanol, 1 x 5 min, PBS 2 x 5 min, 1 x 10 min PBS + 0.05% Tween 20. Antigen retrieval was performed in sodium citrate buffer (10mM sodium citrate in PBS/0.05% Tween 20) using a microwave to maintain 95°C for 25 min, then TMAs were blocked with 2% BSA at 4°C overnight. Following blocking, TMAs were transferred to humidity chambers and incubated for 2 hrs at RT with Alexa-Fluor-488 Wheat Germ Agglutinin (1:100; ThermoFisher, W11261) and Dapi (1µg/mL; Sigma, 268298) diluted in blocking solution, for 1 hour at RT. Cells were then washed in PBS 5 x 5 mins, then mounted with a cleaned glass coverslip (#1.5, 24 mm x 60 mm) in mounting media (Dako; Pathology Products, S3023).

Microscopy

Spinning Disk Confocal Imaging

Imaging was performed on a Nikon Eclipse Ti2 microscope equipped with a Yokogawa CSU-W1 spinning disk scanhead, a Nikon motorized stage with a Nano-Z100 piezo insert (Mad City, Madison, WI), and either a Plan Apo 60x oil 1.49 NA DIC, SR Plan Apo 60x 1.2 NA water or an Apo TIRF 100x oil 1.49 NA DIC Nikon objective lens. Illumination was provided by a Nikon LUNV 6-line laser unit, and images were captured with a Hamamatsu Orca-Flash 4.0 v3 camera. The system was controlled by NIS-Elements software (Nikon). Cells were imaged either on coverslips (#1.5, 22 mm x 22 mm) or in 35 mm glass bottom dishes (FluoroDish) depending on experimental conditions. For the siRNA-based confinement screen, imaging was also performed on a Nikon Eclipse Ti microscope equipped with a Yokogawa CSU-X1 spinning disc scanhead, a Hamamatsu Orca-flash 4.0 v2 camera and a Plan Apo 60x oil 1.4 NA objective. Illumination was provided by a 4-line Agilent MLC400B Monolithic Laser Combiner. The microscope was

similarly equipped with a Nano-Z100 piezo insert (Mad City) and controlled by NIS-Elements software (Nikon). For live cell imaging, cells were incubated with Hoechst 33342 (1:1000; ThermoFisher), ER-Tracker blue-white (30 min at 1 µM; ThermoFisher), or SirDNA (1 µM; Cytoskeleton) at 37°C at 5% CO_2, then washed and resuspended in 1 ml of cell culture media. Maintenance of sample temperature and humidity was performed by a stage-top incubator (Tokai Hit). To image tumor organoids, glass-bottom cell culture dishes were imaged by spinning disc confocal microscopy with a SR Plan Apo 60x 1.2 NA water objective (Nikon). For each organoid, an 8x8 tile was used to capture the full organoid and area of cell invasion and a z-stack was taken at 0.3 µm intervals for each example for later image analysis.

Super-resolution Confocal Imaging
Imaging of confined live cells for super-resolution was performed using a LSM 880 Zeiss confocal microscope equipped with an Airyscan using a Plan-Apo 63x 1.4 NA oil objective. Airyscan image reconstructions were processed in auto strength mode using ZenBlack software (Version 2.3). Additional analysis was performed in ImageJ (NIH).

Two-Photon imaging
Two-photon imaging of whole-mounted 1205Lu melanoma tumors that had been resected from mice was performed using an upright Leica SP8 DIVE (Deep In Vivo Explorer) 2-photon system using a Leica HC Fluotar L VISIR 25x/0.95 water immersion objective lens (working distance 2.4 mm) (Leica Microsystems, Mannheim, Germany). For 2-photon excitation, an infra-red laser, InSight X3 (Spectra-Physics), dual-beam laser with one fixed 1045-nm line and one tunable line from 680 to 1300 nm was used. Sequential excitation at 1045 nm (InSight laser) was used for Hoechst, second harmonic generated intrinsic signal (SHG) from fibrillar collagen, and mScarlet, while a second imaging sequence with an excitation of 950 nm was used for EGFP fluorescence. Acquisition detection was on 4 spectrally tunable HyD detectors (NDDs) and their range was set as follows HyD-NDD1 (420–465 nm) for Hoechst, HyD-NDD2 (517–528 nm) for SHG, HyD-NDD3 (530–560 nm) for EGFP, and HyD-NDD4 (666–685 nm) for mScarlet, respectively.

Transmission Electron Microscopy (TEM)
In situ fixation of cell monolayers on a glass coverslip for TEM was performed using an EM fixative solution (2% glutaraldehyde in 0.1M cacodylate buffer) for 1hr at room temperature, then stored at 4^0C. Sample processing was performed in cacodylate buffer (0.1M, pH7.4), and fixed in osmium (1% in 0.1m cacodylate buffer) for 1hr at room temperature, then dehydrated in a series of alcohol dilutions, infiltrated with epoxy resin (Polyscience) and embedded. Resin blocks were thin sectioned (70 nm) and mounted on 200 mesh copper grids and counterstained with aqueous uranyl acetate (0.5%) and Reynolds lead citrate. Images were acquired with a Hitachi H7600 TEM.

Image analysis

Quantification of NE permeability and rupture during confinement
For all PDMS confinement assays, time-lapse spinning disk confocal movies of cells transiently transfected with relevant plasmids were used to quantify the percentage of events in the following conditions: Nuclear envelope permeability, defined as the rapid

increase in the mCherry-NLS signal in the cytosol of the cell post-confinement, was scored by careful frame-by-frame visual inspection of time-lapse movies. For all conditions, only adherent cells labeled with all relevant fluorescent markers and which remained in the view field both pre- and post-confinement were included in the analysis. Additionally, any cells with a ruptured or permeable NE prior to confinement, or undergoing division, were not included in the quantification. For analysis of NE permeability (mCherry-NLS leakage) and NE rupture (GFP-cGas foci), 10-40 imaging fields were randomly chosen per dish to ensure unbiased coverage of the confined area. To determine a baseline NE integrity percentage, the first 2 time points prior to initiation of cellular confinement, as described above, were analyzed and quantified for total intact nuclei. Following initiation of confinement, images were acquired every 1-2 min for a standard duration of 60 min and quantified again for total intact or ruptured nuclei using the last 2 time points. All movies were analyzed individually and scored based on the total percentage of mCherry-NLS and GFP-cGas positive cells from the sum of all fields of view in each cell culture dish. Any dish containing less than 200 cells was excluded to minimize skewing of data due to low cell density.

For analysis of nuclear integrity in 3D tumor organoids, organoids were generated, imaged and fixed and stained as described above and analyzed using Imaris v9 (Bitplane, Switzerland). First, Imaris surface rendering utilizing the phalloidin channel was used to segment the whole tumor organoid and the core of the tumor organoid was manually segmented, defined as the center of the organoid approximately 200-250 µm from tumor edge. The centroid of this area was used to quantify cell migration distance from the tumor core. Next, using Imaris spot detection and the DAPI channel, individual nuclei were counted in the tumor spheroid edge and invasive periphery and classified as either positive or negative for NE integrity based on the presence or absence of a perinuclear GFP-cGas signal that directly colocalized with the DAPI channel. cGAS foci that were not in contact with a cell nucleus were excluded from the counts. Each organoid was given a percentage score of GFP-cGas foci (cGas positive nuclei/ total nuclei) in each organoid region.

For *ex vivo* tumor analysis, we collected a series of x-y-z images (typically 1 × 1 × 3 μm^3 voxel size) along the *z*-axis at 3 µm intervals over a 5x4 mm region with a range of 100–220 µm z depth throughout the whole-mount tumors, using the tile function (Navigator) of the Leica LAS-X software to automatically generate stitched volumes. For 3D renderings, segmentations and quantitative image analyses, we used Imaris v 9 or 10.0.1 software. Quantification of tumor cell density was performed using the QuPath (20) image analysis software package. Briefly, tumors were first annotated using a semi-automated segmentation, which classified the tumor boundary based on fluorescent intensity from the 1205Lu cells from the surrounding ECM. Next, nuclei were automatically segmented utilizing the Hoechst fluorescent intensity, with the following parameters: (nuclei background radius of 8 µm; filter radius 0; minimal nuclear area 23 μm^2; maximum nuclear area 500 μm^2). These nuclei detections were then used to calculate nuclear features and total number of tumor cells to normalize to the total tumor volume to generate a measure of tumor cell density. Generation of the tumor density maps was calculated with the following parameters: (Density Type: by area (raw counts); density radius 100; range 0-150 cells).

Nuclear Height

Quantification of nuclear height pre- and post-confinement was performed using a spinning disk confocal and a SR Plan Apo 60x 1.2 NA water objective (Nikon). To determine nuclear height, Z-stacks were taken every 0.3 µm pre and post cellular confinement for 3D image reconstruction. Hand-drawn line ROIs were generated using ImageJ (NIH) to measure the approximate Z height of the nucleus.

Nuclear Envelope Width

Analysis of NE spacing was performed using TEM images of immortalized melanocytes (14 cells) and 1205Lu cells (11 cells) using hand drawn ROI's measuring the distance from the INM and ONM over a distance of 200nm at the nuclear periphery. Briefly, using ImageJ (NIH) a 1 pixel line was drawn from the membrane boundary of the INM to the membrane boundary of the ONM when both membranes could be clearly resolved in the image, over a distance of 200nm, resulting in 50-100 measurements per cell.

Nuclear Morphology from Tumor Microarrays

Analysis of nuclear morphology of cells in tumor microarrays of melanoma cancer progression (ME1004F, Biomax) was performed using 5 fields of view taken from each patient sample representing benign nevi (13 samples), stage 1 and 2 (23 samples), stage 3 (4 samples) and lymph node metastasis (11 samples). After immunofluorescence was performed, a custom made ImageJ macro for automatic segmentation of the nucleus based on the DAPI channel using Otsu segmentation, was applied to the images to quantify NE solidity (Area/Convex Area).

Bleb perimeter and area

Quantification of NE bleb morphology was performed from time-lapse movies of cells transiently transfected with GFP-cGas and mCherry NLS. To determine maximum bleb perimeter and area, cells exhibiting NE blebs representing 1205Lu WT (116 cells), and LBR KD (139) were isolated to generate individual fields of view. Using the mCherry-NLS channel for segmentation, ROI's were hand drawn around the frame representing the maximum bleb perimeter and quantified for shape descriptors.

LBR enrichment at the NE

Quantification of NE LBR enrichment was performed from super-resolution Z-stacks of 1205Lu cells either confined or not confined to 3 µm. LBR clusters are defined as heterotypic accumulations of LBR intensity along the NE periphery in unconfined 1205Lu cells with and without LBR depletion. To perform intensity line scan analysis, a 5 pixel width line was drawn at a single plane representing a middle cross section of the nuclear envelope and intensity measurements were recorded and plotted. To determine the variance in LBR signal intensity we utilized the metric of standard deviation over the mean. Additional intensity analysis was performed in confined cells experiencing NE blebbing, where LBR enrichment, defined as an increase in endogenous LBR signal intensity, was determined using a 10 pixel width line, to report on the signal intensity at the NE periphery of both LBR and peripheral heterochromatin.

Analysis of organoid and tumor volume

Analysis of 3D organoid and tumors was performed using samples generated and imaged as described above. For organoids, segmentation was performed utilizing the phalloidin channel, for *ex vivo* tumors the Hoechst channel was used for segmentation. In both samples only cells in the tumor bulk that were not invading into the ECM were

used to calculate 3D volume. Data was then exported to excel and transferred to Prism for statistical analysis.

Atomic force microscopy (AFM)

Immortalized human melanocytes (IM) or 1205Lu melanoma cells expressing either GFP-H2B or GFP-Lap2β (to visualize the nucleus) were plated on glass-bottom dishes (Willco Wells) coated with 10μg/mL of fibronectin. AFM force spectroscopy experiments were performed using a Bruker BioScope Catalyst AFM system mounted on an inverted Axiovert 200M microscope (Zeiss) equipped with a confocal laser scanhead 510 Meta (Zeiss) and a 40x objective lens (0.95 NA, Plan-Apochromat, Zeiss). The microscope system was placed on an acoustic isolation table (Kinetic Systems). During AFM experiments cells were maintained at 37°C using a heated stage (Bruker). A modified AFM microcantilever with an attached 25 μm polystyrene bead (Novascan) was used for all AFM measurements. All AFM microcantilevers were pre-calibrated using the standard thermal noise fluctuations calibration method. The calibrated spring constants were 0.6N/m-1.2N/m. For nuclear measurements, five force curves were performed in succession with a 30 sec delay between each measurement. The applied force was set to be between 20nN – 40nN, yielding indentations between 1.5μm- 3μm, allowing us to press against and deform the cell's nuclei, confirmed by confocal microscopy. The force curves ramp rate was set to 0.5Hz yielding AFM probe approach/compressive speeds between 3μm/s - 5μm/s. The nucleus effective stiffness (N/m) was analyzed and determined using the Bruker NanoScope Analysis software. In brief, force curves were corrected for the non-contact region slope (typically arising from the hydrodynamic drag and AFM probe-sample orientation) using a baseline function. To separate the nuclear mechanical component from the cortical cytoskeleton component, we first utilized sharp AFM probes (PFQNM-LC, Bruker) to determine that the nuclear depth in melanocytes and 1205 Lu cells was approximately 1.5μm in depth after penetrating the plasma membrane. This was further indicated by the bimodal distribution of stiffness on the AFM force curve. Due to this, we selected a region in the force curve ~1.5μm beyond the initial indentation where we then fit a linear regression to extract the nuclear effective stiffness for all experimental conditions.

Measurement of cellular cholesterol

Measurement of total cellular cholesterol was performed using the Amplex Red Cholesterol Assay (Molecular Probes, Thermo Fisher Scientific) per manufacturer's instructions. Briefly, cells were cultured for 7 days in Tu 2% media (1205Lu) supplemented with lipid-depleted FBS (Omega Scientific, Fisher Scientific). Dox-inducible LBR-KD was initiated 3 days prior to the experiment, followed by expression of LBR constructs 24 hours prior to analysis. Directly before the assay, 2000 cells were seeded into each well of a black walled 96 well imaging dish (Thermo Fisher) and incubated for 30 min at 37°C in the presence of 2U/mL HRP, 2 U/mL cholesterol oxidase, 0.2 U/ml cholesterol esterase and 300μM Amplex red reagent. Fluorescence was measured using an iBright 1500 (Thermo Fisher) and quantified using ImageJ. Intensity measurements were determined by first subtracting background fluorescence from a no-cholesterol control well and normalizing each sample to the 1205Lu WT condition.

Depletion of cellular cholesterol

To acutely deplete cellular cholesterol, cells were transiently transfected with mCherry NLS and GFP-cGas and plated overnight. Following labeling cells were washed with PBS and treated with 0.5 mM of MβCD for 15 min at 37°C per published protocols (21). Following treatment cells were confined over 1 hour and NE rupture was quantified as previously described. To inhibit the biosynthesis of endogenous cholesterol, cells were treated with 20μM simvastatin dissolved in DMSO for 48 hours prior to any experimental analysis and confined as previously described.

GC-MS for sterol content

Prior to lysate collection, cells were cultured for 7 days in Tu 2% media (1205Lu) or dermal cell basal media (melanocytes) and supplemented with lipid-depleted FBS (Omega Scientific, Fisher Scientific). LBR knockdown was induced through dox addition as described above and confirmed by western blot analysis. Cell lysates were collected from pellets of ~ $2x10^6$ cells and stored at -80°C prior to analysis. Sterol analysis was performed using ion-ratio GC/MS using either an Agilent DB-5MS-30m x 0.25mm x 0.25μm column or a Phenomenex ZB-50 30m x 0.32mm x 0.25μm column, on an Agilent 6390N/5973 GC/MS system as previously described (22) with modifications to the GC/MS method to include ions for additional intermediates in the cholesterol biosynthetic pathway between lanosterol and cholesterol. Lymphoblasts from a patient with a non-lethal skeletal dysplasia resulting from compound heterozygous variants in LBR similar to that seen in cells from patients with Greenberg dysplasia were used as a positive control for LBR deficiency (23).

Western blot analysis

Cells were lysed in Laemmli sample buffer, separated by SDS-PAGE and electro-transferred at room temp (RT) for 1 hour to Immobilon-P PVDF membrane. Membranes were blocked for 1 hour at RT with 5% nonfat dry milk (wt/vol) in TBS-T buffer (TBS + 0.1% Tween-20 [vol/vol]), incubated for 2 h at RT with indicated primary antibodies, washed 3 x 5 min in TBS-T, incubated with appropriate HRP-conjugated secondary antibodies (1:10,000) for 1h at RT, then washed 3 x 5 min in TBS-T. An ECL detection system (Millipore) was used to visualize protein bands. The following antibodies were used: anti-LBR (1:500; Abcam ab32535), anti-lamin A/C (1:1000; Abcam, ab108595), rabbit anti-lamin B1 (1:1000; Abcam, ab16048), mouse anti-lamin B2 (1:1000; Abcam, ab8983), rabbit anti-GAPDH (1:5000; Clone 14C10, Cell Signaling, 2118S), anti-TM7SF2 (1:500; Proteintech), anti-mCherry (1:1000; Abcam ab167453). The secondary antibodies (HRP-conjugated goat anti-mouse (115-035-003) or goat anti-rabbit (111-035-003)) were from Jackson ImmunoResearch Laboratories. Quantitative analysis was achieved by first performing local background subtraction around bands of interest, then calculating the ratio between the band of interest and the loading control (GAPDH). Band intensities were normalized to WT levels to obtain percent relative to WT using ImageJ (NIH).

Statistical analysis

Statistical analysis was performed in Prism (GraphPad Software version 9). Graphs represent mean values± SEM or ± SD as indicated in the figure legends of at least three independent experiments. Statistical significance was obtained using ANOVA or student t-test with Welch's, Kolmogorov-Smirnov or Mann-Whitney correction as indicated in the figure legends. Significance is reported as *p ≤ 0.05, ** p ≤ 0.01, ***p ≤ 0.001, and ****p ≤ 0.0001.

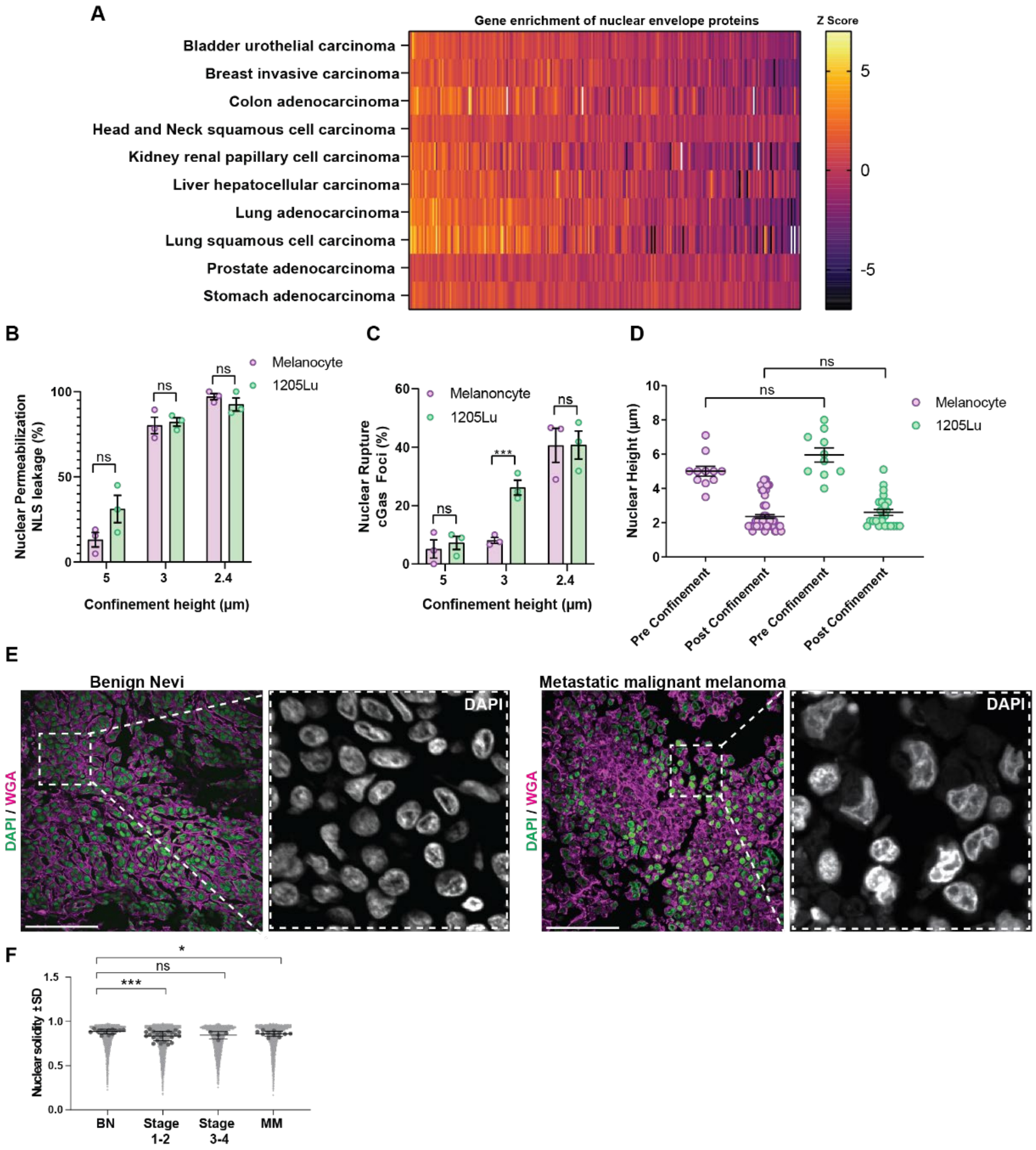

Fig. S1.

(A) Heat map of transcript abundance (normalized relative to a baseline human genome) of 249 genes encoding nuclear envelope proteins (curated from the Human Protein Atlas) from RNA-seq data from 6860 clinical samples representing 10 cancer subtypes from The Cancer Genome Atlas (TCGA) PanCancer repository. **(B, C)** Quantification of NE permeabilization (B, left) and NE rupture (C, right) in human melanocytes (purple) and 1205Lu cells (green) at different confinement heights (Melanocytes n=3 experiments for all heights, n=404 cells (2.4μm), n=196 cells (3μm), n=184 cells (5μm); 1205Lu n=3 experiments for all heights, n=301 cells (2.4μm) n=604 cells (3μm), n=1375 cells (5μm). **(D)** Nuclear height of fixed 1205Lu cells (green) and human melanocytes (purple) expressing GFP-NLS measured from confocal image series before (Pre Confinement)

and after (Post Confinement) confinement to 3µm. 1205Lu: before confinement n=3 experiments, 189 cells; after confinement n=3 experiments, 159 cells. Human melanocytes: before confinement n=3 experiments, 107 cells; after confinement n=3 experiments, 71 cells. **(E)** Confocal images of tissue microarray sections of human clinical samples of benign nevi (left 2 panels) and metastatic melanoma (right 2 panels), DNA stained with DAPI (left, green, and right greyscale) and the plasma membrane stained with Alexa 488 wheat germ agglutinin (WGA, left, purple). Dotted boxed region zoomed at right. Bar= 100µm. **(F)** Quantification of solidity (area/convex hull area) of nuclei in DAPI-stained tissue microarray samples from benign nevi (BN, n= 3099 nuclei in 3 tissue samples), stage 1 and 2 tumors (Stage 1-2, n= 3612 nuclei in 6 tissue samples), stage 3 and 4 tumors (Stage 3-4, n= 1095 nuclei in 3 tissue samples), and malignant melanoma (MM, n= 1055 nuclei in 3 tissue samples). Grey points=individual nuclei, black points= mean of each tissue sample Data points represent independent experiments each consisting of pooled cell data from a single imaging dish (B,C) or individual cells (D, F). (B,C,D) Significance was tested with a Student's T-test Mann-Whitney, bar= mean, error bars= SEM; (F) Bar= mean; boxes= 25th to 75th percentile.

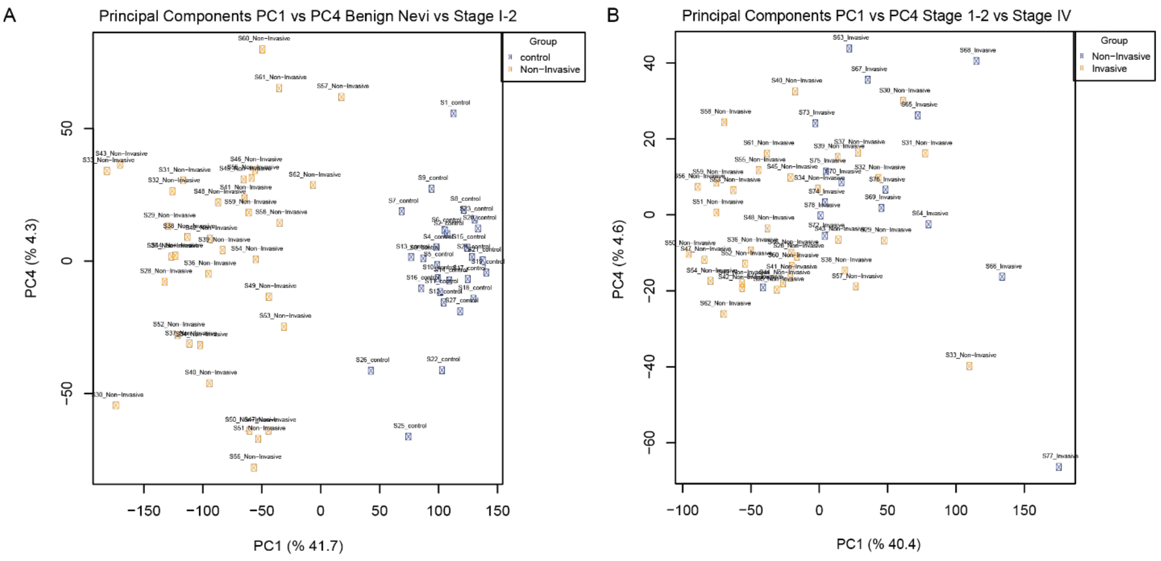

Fig. S2.
(A,B) Principle component analysis (PCA) of differentially expressed genes from RNA-seq datasets (GEO:GSE98394) showing pairwise comparison by clinical stage. Separation between groups in PCA analysis represents distinct patterns of gene expression. (A) comparison between benign nevi (control) and stage 1-2 (Non-invasive); **(B)** Stage 1-2 (Non-invasive) versus Stage 3-4 melanoma (Invasive).

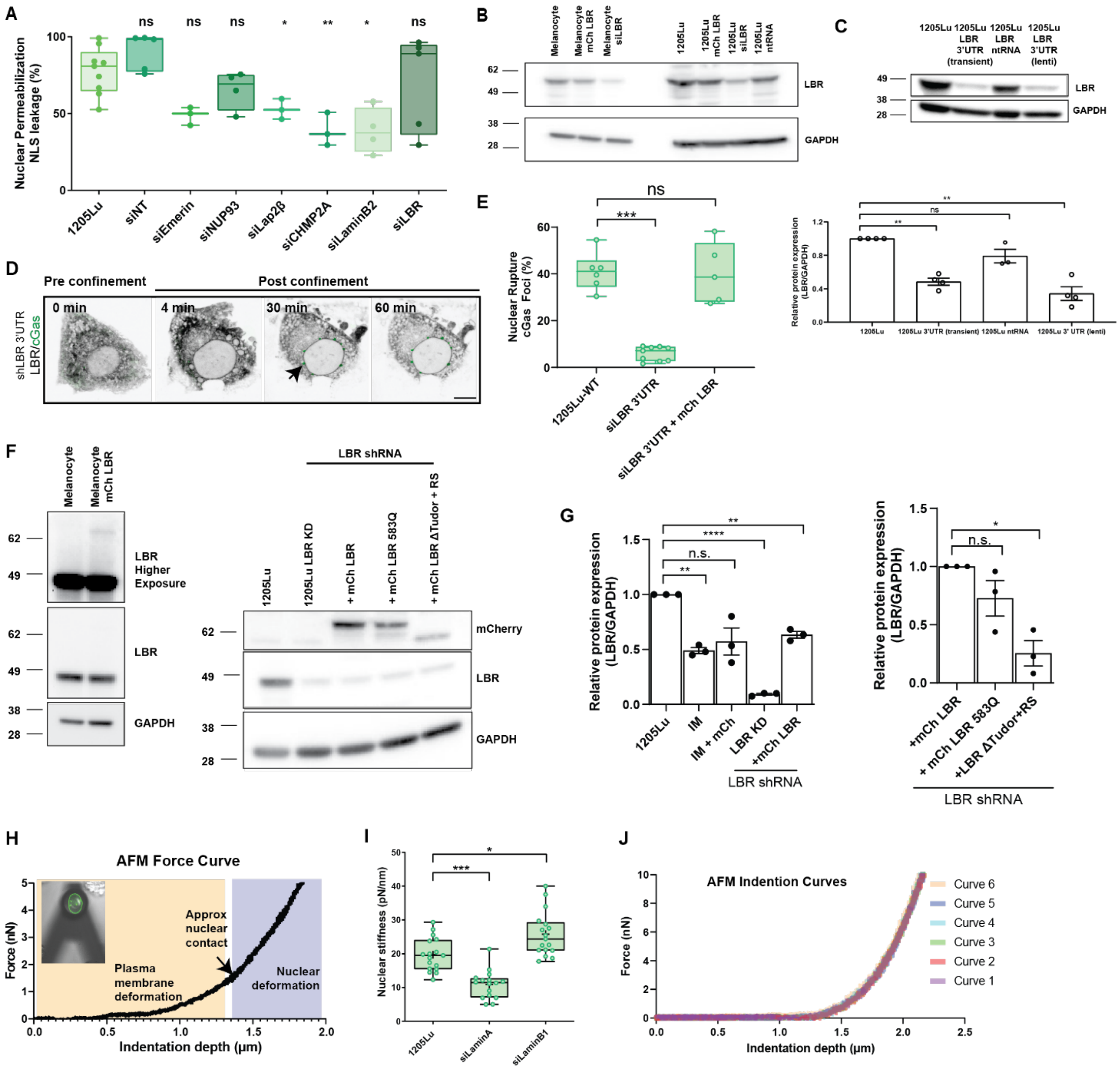

Fig. S3.
(A) Quantification of the fraction of confined cells exhibiting NE permeabilization, as indicated by mCherry-NLS leakage from the nucleus to the cytoplasm in 1205Lu cells with or without (1205Lu, n=9 experiments, 2473 cells) co-transfection with non-targeting siRNA (SiNT, n=5 experiments, 421 cells) or siRNA targeting emerin (siEmerin, n=3 experiments, 261 cells), NUP93 (siNUP93, n=4 experiments, 521 cells), Lap2β (siLap2β, n=3 experiments, 195 cells), CHMP2A (siCHMP2A, n=3 experiments, 204 cells), LaminB2 (siLaminB2, n=4 experiments, 607 cells), or LBR (siLBR, n=5 experiments, 1627 cells). **(B)** (Left) Western blot of lysates of melanocytes with (melanocyte mCh LBR) or without (melanocyte) expression of mCherry LBR or shRNAs targeting the 3’UTR of LBR (Melanocyte siLBR). (Right) Western blot of lysates of 1205Lu cells without (1205Lu) or with expression of mCherry LBR (1205Lu mCh LBR), shRNA

targeting the 3'UTR of LBR (1205Lu siLBR) or non-targeting shRNAs (1205Lu ntRNA). **(C)** (Top) Western blot of lysates of 1205Lu cells without (1205Lu) or with transient expression of shRNA targeting the 3'UTR of LBR (1205Lu LBT 3'UTR (transient)) or NT shRNA (1205Lu LBR ntRNA) or lentiviral-based expression of shRNA targeting the 3'UTR of LBR (1205Lu LBT 3'UTR lenti). (Bottom) Quantitative analysis of experiments like that presented in the panel above normalized by WT-1205Lu LBR level (n=4 independent experiments in each condition). **(D)** Confocal image series of a 1205Lu cell treated with shRNA 3'UTR-mediated LBR depletion, co-transfected with mCherry-LBR (inverted greyscale) and GFP-cGas (green) before (pre confinement) and after (post confinement) confinement to 3 µm. Arrow highlights example GFP-cGas foci, time after initiation of imaging noted. **(E)** Quantification of the fraction of confined cells exhibiting NE rupture in 1205Lu cells with or without (1205Lu, n= 3 experiments, 152 cells) co-transfection with 3'UTR siRNA (siLBR 3'UTR, n= 3 experiments, n=3668 cells) or 3'UTR siRNA together with mCherry LBR (siLBR 3'UTR + mCherry LBR, n= 3 experiments, 1167 cells. **(F)** (Left) Western blot of lysates of melanocytes with (melanocyte mCh LBR) or without (Melanocyte) expression of mCherry LBR (Right) Western blot of lysates of 1205Lu cells without (1205Lu) or with expression of lentiviral-based expression of shRNA targeting the 3'UTR of LBR (1205Lu LBR KD), and full length mCherry LBR (mCh LBR), LBR 583Q point mutation (mCH LBR 583Q) or the N-terminal truncated form mCherry-LBRΔTudor+RS (mCh-LBRΔTudor+RS). Note: The the N-terminal truncation removes the LBR antibody recognition site. **(G)** (Left) Quantitative analysis of experiments like that presented in panel (F) normalized by WT-1205Lu LBR level (n=3 independent experiments in each condition). (Right) Quantitative analysis of experiments like that presented in panel (F) normalized by WT-1205Lu mCherry-LBR level (n=3 independent experiments in each condition). **(H)** AFM force vs displacement curve (plasma membrane and cortex deformation (peach) and nuclear deformation (lavender). Inset: Image of a bead-affixed AFM cantilever resting on the nucleus of a1205Lu cell expressing GFP-Lap2β (green). **(I)** AFM quantification of nuclear stiffness in 1205Lu cells (n=7, n=43 cells), depleted for lamin A (n=3, n=16 cells), or lamin B1 (n=3, n=16 cells) **(J)** Example repeated force curve replicates performed on a single cell. In (D) bar = 10µm. In (A,E) points= means of independent experiments, in (C,G) points= individual experiments, in (I) individual cells. Significance was tested with a Student's T-test Mann-Whitney (A,E), a paired students T-test (C,G), or (I) a student T-test using Welch's correction. bar= mean; error bars, SEM (A,E,I), bar= mean; error bars, min and max .

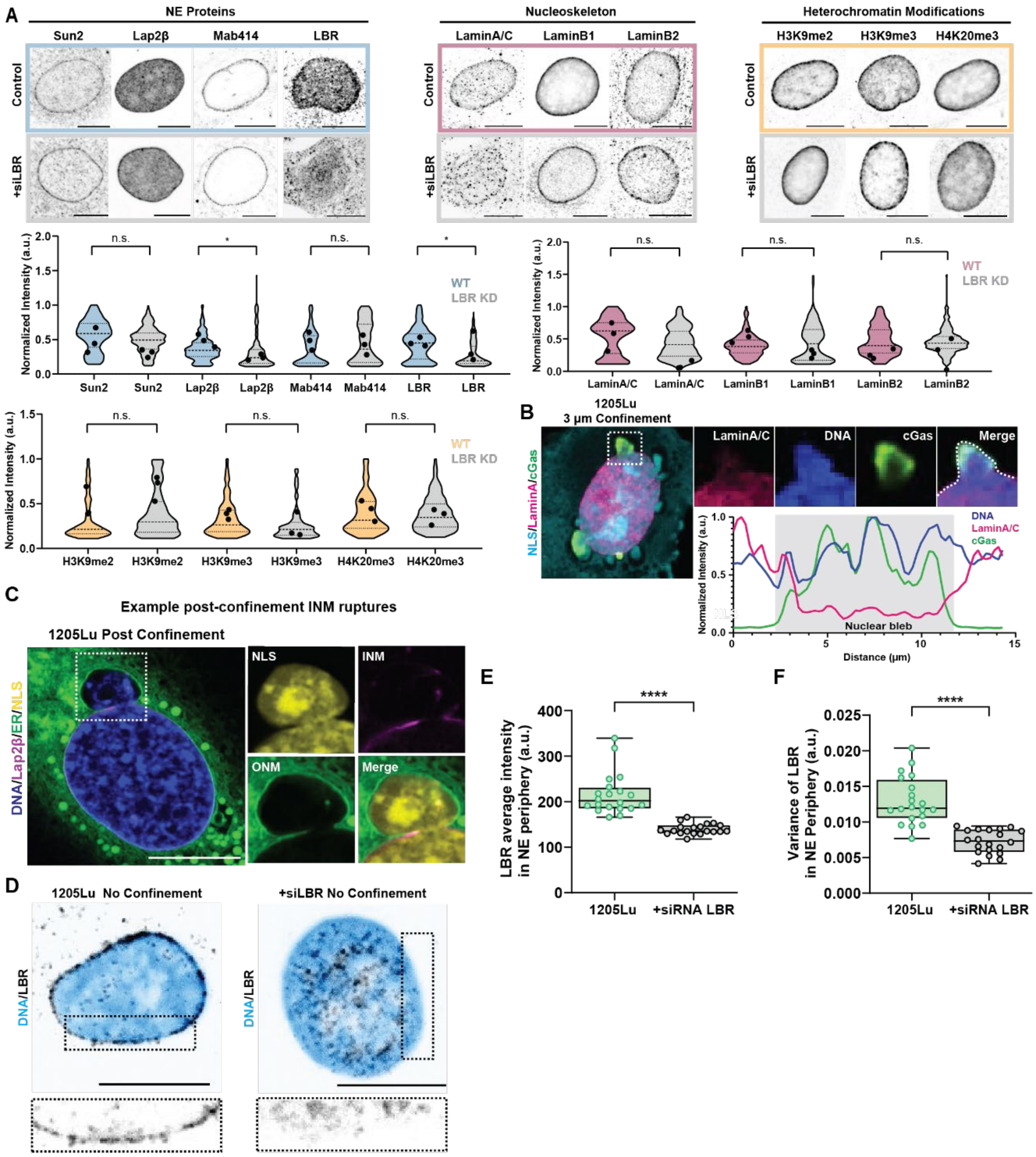

Fig. S4.

(A)(Top) Confocal images of 1205Lu cells with (grey, +siLBR bottom rows) or without (top rows) transfection with siRNAs targeting LBR that were fixed and immunostained for NE proteins (left panel, blue, Sun2, Lap2β, nuclear pores (Mab414) and LBR), the nucleoskeleton (middle panel, dusky rose, lamin A/C, lamin B1, lamin B2) and heterochromatin modifications (right panel, melon, H3K9me2, H3K9me3, H4K20me3). (Below) Quantification of immunostaining at the NE. Sun2: WT n=3 experiments, 198 cells; siLBR n=3 experiments, 242 cells. Lap2β: WT n=3 experiments, 265 cells; siLBR n=3 experiments, 216 cells. Nuclear pores (Mab414): WT n=3 experiments, 242 cells; siLBR n=3 experiments, 116 cells. LBR: WT n=3 experiments, 268 cells; siLBR n=3 experiments, 173 cells. Lamin A/C: WT n=3 experiments, 112 cells; siLBR n=3

experiments, 175 cells. Lamin B1: WT n=3 experiments, 138 cells; siLBR n=3 experiments, 120 cells. Lamin B2: WT n=3 experiments, 273 cells; siLBR n=3 experiments, 220 cells. H3K9me2: WT n=3 experiments, 165 cells; siLBR n=3 experiments, 169 cells. H3K9me3: WT n=x3 experiments, 227 cells; siLBR n=3 experiments,108 cells. H4K20me3: WT n=3 experiments, 138 cells; siLBR n=3 experiments, 114 cells. **(B)** Confocal image of a 1205Lu melanoma cell transfected with GFP-cGas (green) and mCherry NLS (cyan) that was fixed during confinement to 3µm and stained with SiR DNA (blue) and immunostained for Lamin A/C (magenta). Zoom of boxed region (above, right), intensity linescan along dotted line (below, right). **(C)** Super-resolution confocal images during confinement to 3 µm of living 1205Lu cells transfected with mCherry-NLS (yellow) and GFP-Lap2β (magenta) and stained with blue-white ER-Tracker (green) and SiR DNA (blue). Zooms of white dashed boxed regions (right). **(D)** Confocal images of 1205Lu cells with (right, +siLBR bottom rows) or without (left) transfection with siRNAs targeting LBR that were fixed and immunostained for LBR (grey) and Hoechst (blue). Zoom of boxed region (bottom). **(E, F**) Quantification of LBR intensity from the NE periphery from images such as those in **(D)**. **(E)** Average intensity at the NE periphery. **(F)** Variance of intensity across NE periphery (1205Lu: n=4 experiments, 20 cells; +siRNA: n=3 experiments, 20 cells). In (A, B, C) bar = 10µm. In (A), data points represent means of individual experiments and significance was tested with a Student's T-test Welch,bar= mean, in (E, F) data points represent individual cells, and Significance was tested with a student's T-test Mann-Whitney, bar=mean, error bars min and max.

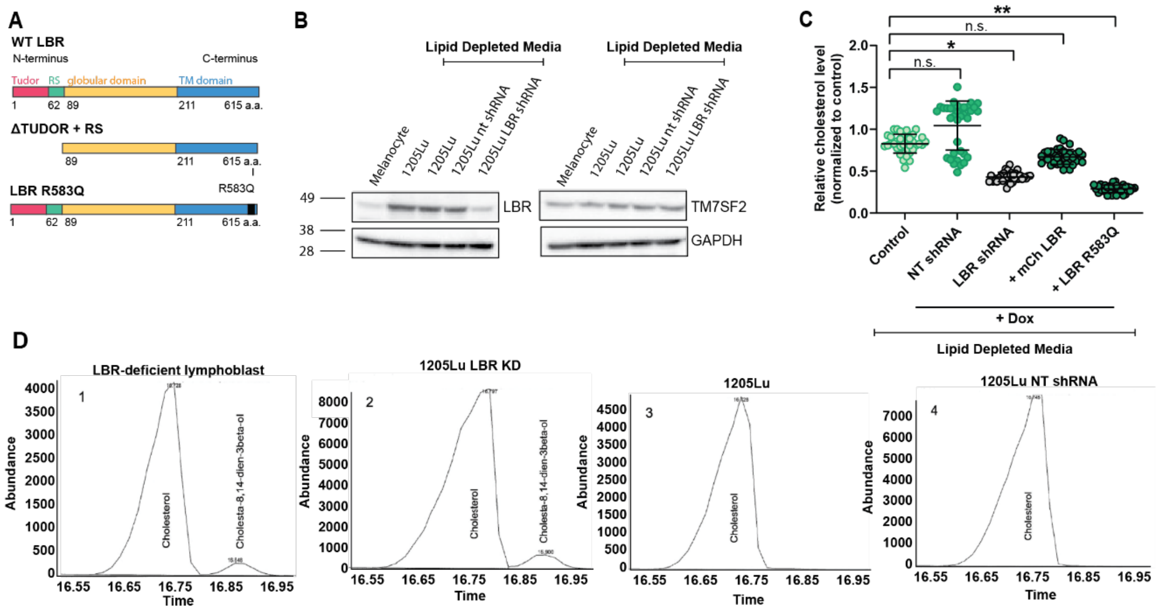

GC-MS Sterol Measurement

Cell Line	Cholesterol (µg/mg protein)	cholesta-8,14 dien-3β-ol (% total sterol)	Zymostenol (% total sterol)	Desmosterol (% total sterol)	7-dehydro cholesterol (% total sterol)	Lathosterol (% total sterol)	4αmethyl-5α cholesta-8 en-3β-ol (% total sterol)	4αmethyl-5α cholesta-7 en-3β-ol (% total sterol)	Dihydro-lanosterol (% total sterol)	Lanosterol (% total sterol)	4,4-dimethyl-5α cholesta-8 en-3β-ol (% total sterol)	4,4-dimethyl5α cholesta-8(9) 24-dien-3β-ol (% total sterol)	Total Sterols
1205Lu	23.13	0.00	0.19	0.25	0.04	0.55	0.01	0.00	0.00	0.02	0.00	0.00	23.43
1205Lu NT shRNA	19.79	0.00	0.11	0.35	0.03	0.46	0.01	0.00	0.00	0.02	0.00	0.00	20.02
1205Lu LBR shRNA	20.40	0.28	0.18	0.27	0.08	0.46	0.02	0.00	0.00	0.04	0.00	0.00	20.75

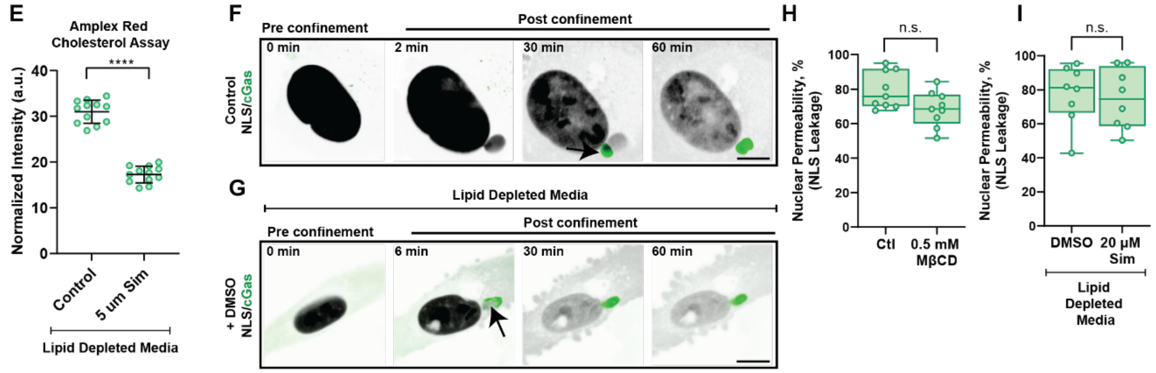

Fig. S5. (A) Schematic diagram of LBR functional domains and mutants. **(B)** Western blot showing levels of TM7SF2 and LBR in melanocytes, 1205Lu, and 1205Lu treated with either non-targeting shRNAs (nt shRNA) or targeting the 3'UTR of LBR (LBR shRNA) cultured for 7 days in either normal or lipid depleted media. **(C)** Values of total sterols and sterol intermediates from GC-MS in 1205Lu, NT shRNA and LBR-KD cultured for 7 days in lipid depleted media**.** Red text indicates the percentage of cholesta-8,14-dien-3β-ol accumulation out of total cellular sterols. **(D)** Spectrophotometric determination of cholesterol level in lysates of 1205Lu treated with DMSO (Vehicle control) or with 5µM simvastatin. **(E)** Quantification of the fraction of

confined cells exhibiting NE permeabilization in 1205Lu cells treated with vehicle (+DMSO, n=3 experiments, 443 cells) or 5 µM (n=3 experiments, 1182 cells) or 20 µM (n=3 experiments, 559 cells) simvastatin and expressing GFP-cGas and mCh-NLS. **(F,G)** Confocal image series of cholesterol depletion control experiments where 1205Lu cells are treated with either 2% Tu media (Control) or vehicle (+DMSO) and expressing mCherry-NLS (inverted grayscale) and GFP-cGas (green), before and after confinement to 3 µm. Arrow highlights cGas focus. **(H)** Quantification of the fraction of confined cells exhibiting NE permeability in 1205Lu cells in media (Ctl, n=3 experiments, 738 cells) or treated with 0.5 mM MβCD (n=3 experiments, 1329 cells) and expressing GFP-cGas **(I)** Quantification of the fraction of confined cells exhibiting NE rupture in 1205Lu cells treated with vehicle (+DMSO, n=3 experiments, 443 cells) or 20µM (n=3 experiments, 559 cells) simvastatin and expressing GFP-cGas. In (F, G) bar = 10 µm. Data points represent means of individual experiments (H,I) n= 3 experiments**.** Significance was tested with (C) ANOVA , bars= mean; error bars=SD, (E,H,I) Student's T-test with Kolmogorov-Smirnov correction, error bars= min and max, and boxes= 25th to 75th percentile.

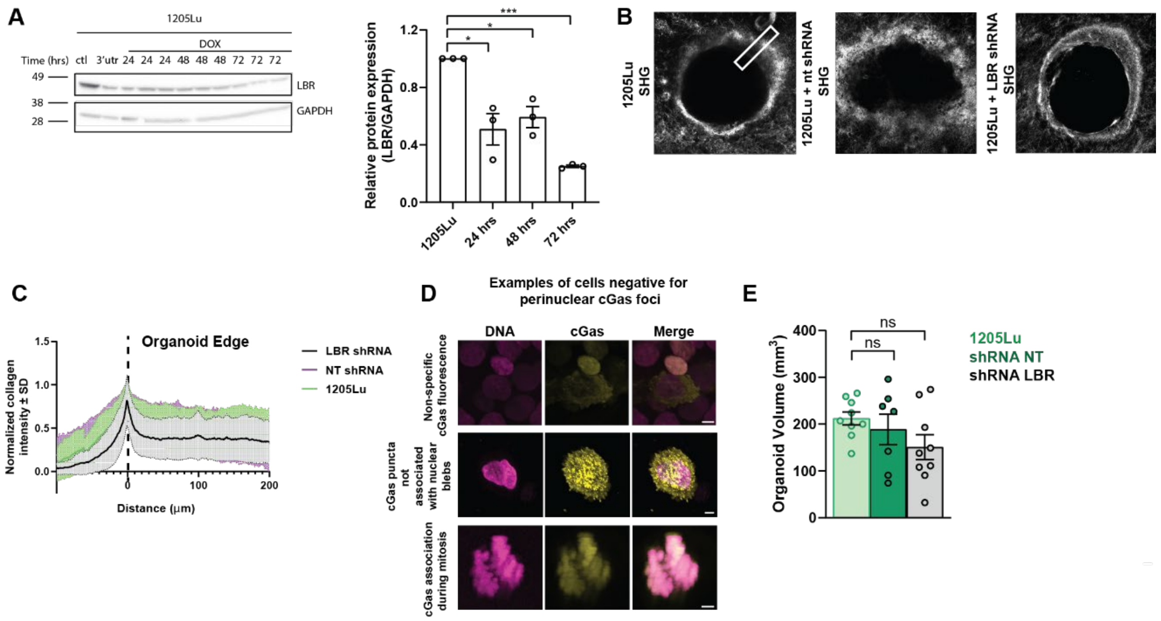

Fig. S6.
(A) Western blot of doxycycline-induced stable knockdown of LBR (left) and quantification (Right). **(B)** 3D projections of z-series of second harmonic images of collagen in organoids grown from 1205Lu cells with either (shRNA LBR, n=3 organoids) or (NT shRNA, n=3 organoids) or without (1205Lu, n=3 organoids). Box indicates an example of a region for line scananalysis presented in (C). **(C)** Quantification of collagen intensity from (B) Dashed line at the x-axis origin indicates edge of organoid, color fill represents standard deviation (SD) from the mean of collagen intensity. **(D)** Representative example images of cells from organoids stably expressing GFP-cGAS (yellow) and mScarlet-NLS (magenta) quantified as negative for perinuclear cGas foci (bar = 5μm). **(E)** Quantification of organoid volume from 3D reconstructions of z-series of confocal images. Significance was tested with a paired student's T-test (A); bars=mean, error bars with SEM. In (E) significance was tested with a Student's T-test Welch correction, bars= means with SEM.

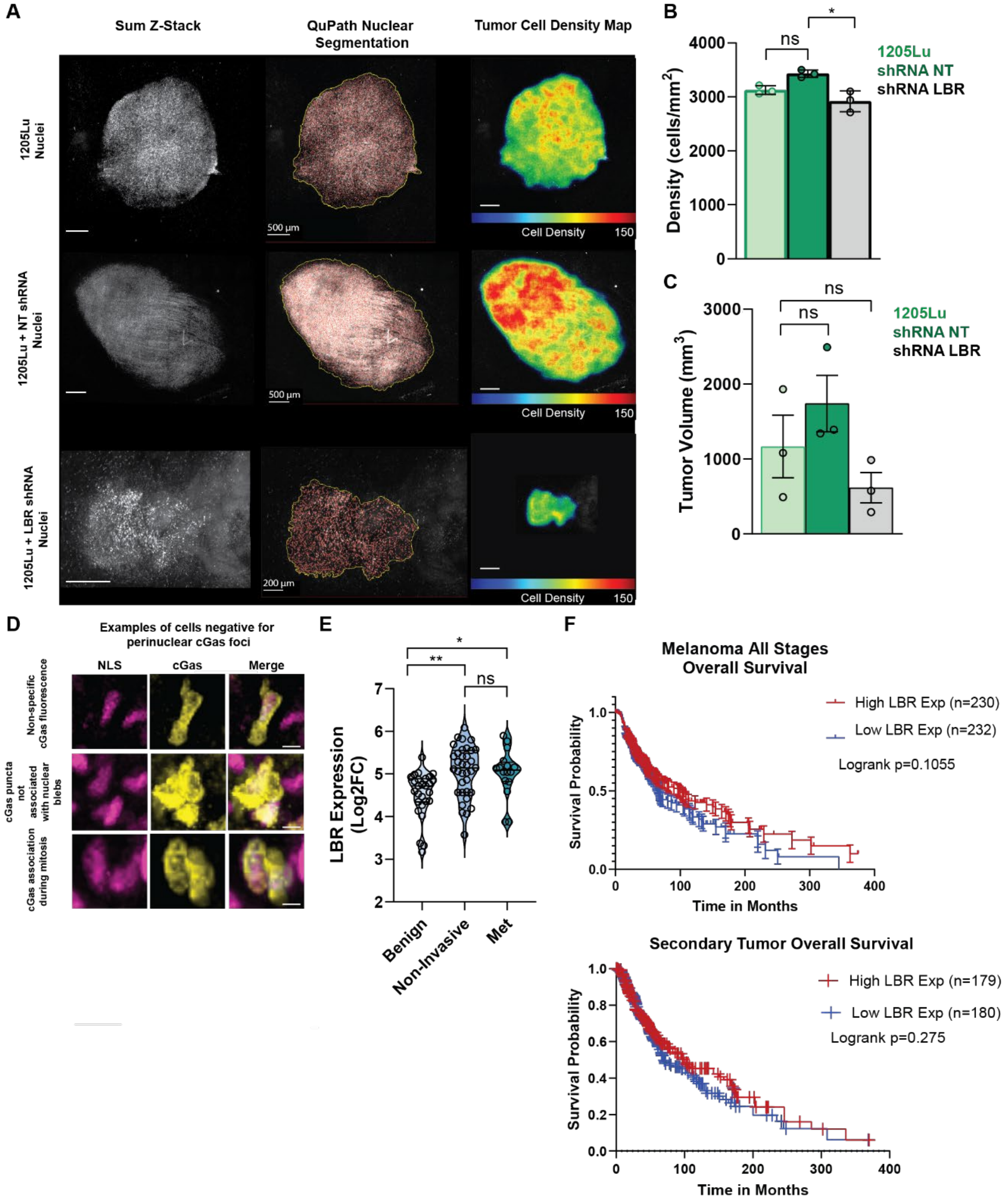

Fig. S7.

(**A**) Representative nuclear segmentation performed using QuPath. (Left) a sum slice Z-stack of the NLS channel was used to segment the nuclei. (Middle) Representative QuPath tumor boundary annotation (yellow line) and nuclear detection (red). (Right) Tumor cell density maps color coded by cells per mm^2. For 1205Lu and 1205Lu NT bar = 500 µm, for LBR shRNA bar = 200 µm. **(B,C)**Quantification of tumor cell density (B) and tumor volume (C) from 3D reconstructions of z-series of two photon images using Imaris

for reconstruction. **(D)** Representative example images of cells from melanoma tumors stably expressing GFP-cGAS (yellow) and mScarlet-NLS (magenta) quantified as negative for perinuclar cGas foci (bar = 5μm). **(E)** LBR Log2FC from RNA-seq data from individual patient biopsies of benign nevi (Benign), stage I-II (Non-Invasive) and metastatic melanoma (Met) tumors. **(F)** Kaplan-Meier survival probability for SKCM patients from TCGA (Top), or Secondary Tumor Overall Survival (Bottom) color coded by LBR expression level in all tumor stages. (B,C) Significance was tested with a Student's T-test Welch correction, bars= means with SEM, in (E) ANOVA. (F) Significance was tested with log-rank.

Table S1.

Table of expression levels of bioinformatically annotated “NE proteins” across cancer subtypes, taken from the TCGA database and portrayed in Fig. S1A.

Movie S1.

Confocal image series of benign and malignant cells before and after confinement to 3µm. Representative cell lines for skin (immortalized human melanocytes, 1205Lu), breast (MCF10A or T47D) and prostate (RWPE-1, PC-3) transfected with mCherry-NLS (inverted grayscale) and GFP-cGas (green). Arrows highlight GFP-cGas foci indicating nuclear rupture. Time interval between images 60 s. Scale bar, 10µm.

Movie S2.

Confocal image series of 1205Lu cells transfected with mCherry-NLS (inverted grayscale) and GFP-cGas (green) with and without treatment with the following siRNA’s to specifically deplete NE proteins; 1205Lu WT (top, left), nt siRNA (top, center left), emerin (top, center right), NUP93 (top right), Lap2b (bottom left), CHMP2A (bottom center left), laminB2 (bottom center right) or LBR (bottom right), before and after confinement to 3mm. Arrows highlight GFP-cGas foci indicating nuclear rupture. Time interval between images 120 s. Scale bars, 10µm.

Movie S3.

Confocal image series of immortalized human melanocytes transfected with mCherry-NLS or mCherry-LBR (inverted grayscale) and GFP-cGas (green) before and after confinement to 3µm. Arrows highlight GFP-cGas foci indicating nuclear rupture. Time interval between images 120 s. Scale bar, 10µm.

Movie S4.

Superresolution Z-series of a 1205Lu transfected with EGFP- Lap2b (magenta), and vital dyes blue-white ER tracker (green) and siR-DNA (blue) after confinement to 3mm, highlighting differential rupture of the INM and ONM. Scale bar as shown in movie. Superresolution Z-series of a 1205Lu transfected with siRNA depleting LBR, EGFP-Lap2b (magenta), and vital dyes blue-white ER tracker (green) and siR-DNA (blue) after confinement to 3mm, highlighting differential rupture of the INM and ONM. Scale bar as shown in movie.

Movie S5.

Confocal image series of 1205Lu cells transfected with GFP-cGas (green) and treated and siRNA targeting the 3’UTR of LBR (+LBR siRNA) together with either mCherry-LBR (left, inverted grayscale), mCherry-LBRDTudor+RS (left center, inverted grayscale), mCherry LBR 583Q (right center, inverted grayscale) or mCherry LBR 1402DT (right end, inverted grayscale), before and after confinement to 3 µm. Arrows highlight GFP-cGas foci indicating nuclear rupture. Time interval between images 120 s. Scale bar, 10 µm.

Movie S6.

Confocal image series of 1205Lu cells transfected with mCherry-NLS (inverted grayscale) and GFP-cGas (green) treated with either vehicle control (2% Tu Media) or 0.5 mM MβCD, before and after confinement to 3 µm. Arrow indicates cGas focus,

indicating a cell positive for nuclear rupture. Time interval between images 120 s. Scale bar, 10 μm.

Movie S7.

Confocal image series of 1205Lu cells transfected with mCherry-NLS (inverted grayscale) and GFP-cGas (green) treated with either vehicle control (DMSO) or 20 μM simvastatin, before and after confinement to 3mm. Arrow indicates cGas focus, indicating a cell positive for nuclear rupture. Time interval between images 120 s. Scale bar, 10 μm.

Movie S8.

3D reconstruction of a 17.5μM-thick confocal Z-series of fixed tumor organoids grown from 1205Lu cells stably expressing GFP-cGas (yellow) and mScarlet-NLS (magenta) in a collagen ECM. Scale bar as shown in movie.

Movie S9.

3D reconstruction of a 36μM-thick confocal Z-series of fixed tumor organoids grown from 1205Lu cells stably expressing GFP-cGas (yellow), mScarlet-NLS (magenta) and shRNA targeting LBR, in a collagen ECM. Scale bar as shown in movie.

Movie 10.

3D reconstruction of a 223μM-thick two-photon confocal Z-series of living ex-vivo tumors grown from 1205Lu cells stably expressing GFP-cGAS (yellow) and mScarlet-NLS (magenta) in mouse dermis. Scale bar as shown in movie. Gamma correction was applied to SHG channel (greyscale) to allow for better visualization of dim collagen fibers.

Movie S11.

3D reconstruction of a 145μM-thick two-photon confocal Z-series of living ex-vivo tumors grown from 1205Lu cells stably expressing GFP-cGAS (yellow), mScarlet-NLS (magenta) and shRNA targeting LBR in mouse dermis. Scale bar as shown in movie. Gamma correction was applied to SHG channel (greyscale) to allow for better visualization of dim collagen fibers.

References

1. H. R. Thiam, *et al.*, NETosis proceeds by cytoskeleton and endomembrane disassembly and PAD4-mediated chromatin decondensation and nuclear envelope rupture. *Proceedings of the National Academy of Sciences* **117**, 7326–7337 (2020).

2. H. R. Thiam, *et al.*, NETosis proceeds by cytoskeleton and endomembrane disassembly and PAD4-mediated chromatin de-condensation and nuclear envelope rupture. [Preprint] (2019). Available at: https://www.biorxiv.org/content/10.1101/663427v1 [Accessed 26 August 2025].

3. S. Wee, *et al.*, PTEN-deficient cancers depend on PIK3CB. *Proceedings of the National Academy of Sciences* **105**, 13057–13062 (2008).

4. E. Becht, *et al.*, Dimensionality reduction for visualizing single-cell data using UMAP. *Nat Biotechnol* (2018). https://doi.org/10.1038/nbt.4314.

5. A subcellular map of the human proteome | Science. Available at: https://www.science.org/doi/10.1126/science.aal3321 [Accessed 18 December 2025].

6. K. A. Hoadley, *et al.*, Cell-of-Origin Patterns Dominate the Molecular Classification of 10,000 Tumors from 33 Types of Cancer. *Cell* **173**, 291-304.e6 (2018).

7. B. Badal, *et al.*, Transcriptional dissection of melanoma identifies a high-risk subtype underlying TP53 family genes and epigenome deregulation. *JCI Insight* **2**, 1–15 (2017).

8. I. Tirosh, *et al.*, Dissecting the multicellular ecosystem of metastatic melanoma by single-cell RNA-seq. *Science* **352**, 189–196 (2016).

9. J. Barretina, *et al.*, The Cancer Cell Line Encyclopedia enables predictive modeling of anticancer drug sensitivity. *Nature* **483**, 603–607 (2012).

10. S. W. Wingett, S. Andrews, FastQ Screen: A tool for multi-genome mapping and quality control. *F1000Research* **7**, 1338 (2018).

11. D. A, *et al.*, STAR: ultrafast universal RNA-seq aligner. *Bioinformatics (Oxford, England)* **29**, 15–21 (2013).

12. Y. Liao, G. K. Smyth, W. Shi, FeatureCounts: An efficient general purpose program for assigning sequence reads to genomic features. *Bioinformatics* **30**, 923–930 (2014).

13. L. CW, C. Y, S. W, S. GK, voom: Precision weights unlock linear model analysis tools for RNA-seq read counts. *Genome biology* **15** (2014).

14. Y. G, W. LG, H. Y, H. QY, clusterProfiler: an R package for comparing biological themes among gene clusters. *Omics : a journal of integrative biology* **16**, 284–287 (2012).

15. S. K, *et al.*, Misregulation of ELK1, AP1, and E12 Transcription Factor Networks Is Associated with Melanoma Progression. *Cancers* **12** (2020).

16. B. L. Aken, *et al.*, Ensembl 2017. *Nucleic Acids Res* **45**, D635–D642 (2017).

17. S. X. Ge, D. Jung, R. Yao, ShinyGO: a graphical gene-set enrichment tool for animals and plants. *Bioinformatics* **36**, 2628–2629 (2020).

18. B. T. Sherman, *et al.*, DAVID: a web server for functional enrichment analysis and functional annotation of gene lists (2021 update). *Nucleic Acids Res* **50**, W216–W221 (2022).

19. M. Krishnamurthy, *et al.*, Metastatic organotropism in small cell lung cancer. [Preprint] (2025). Available at: https://www.biorxiv.org/content/10.1101/2024.10.07.617066v2 [Accessed 26 August 2025].

20. P. Bankhead, *et al.*, QuPath: Open source software for digital pathology image analysis. *Sci Rep* **7**, 16878 (2017).

21. S. Mahammad, I. Parmryd, Cholesterol homeostasis in T cells. Methyl-β-cyclodextrin treatment results in equal loss of cholesterol from Triton X-100 soluble and insoluble fractions. *Biochimica et Biophysica Acta (BBA) - Biomembranes* **1778**, 1251–1258 (2008).

22. R. I. Kelley, Diagnosis of Smith-Lemli-Opitz syndrome by gas chromatography/mass spectrometry of 7-dehydrocholesterol in plasma, amniotic fluid and cultured skin fibroblasts. *Clin Chim Acta* **236**, 45–58 (1995).

23. E. Thompson, *et al.*, Lamin B receptor-related disorder is associated with a spectrum of skeletal dysplasia phenotypes. *Bone* **120**, 354–363 (2019).
