# Supplementary material for: Lamin B receptor upregulation in metastatic melanoma causes nuclear envelope fragility in confined migration during cancer invasion
Source: Proc Natl Acad Sci U S A. 2026 Feb 18;123(8):e2513031123. doi: 10.1073/pnas.2513031123 (PMC12933115; doi:10.1073/pnas.2513031123)
Supplement: Supplementary file 2 — Dataset S01 (PDF) [file pnas.2513031123.sd01.pdf]

Table S1

Cancer Sul	BLCA	BRCA	COAD	HNSC	KIRP	LIHC	LUAD	LUSC	PRAD	STAD
CENPF	1.388021	2.763739	1.869111	1.186254	2.430447	3.461051	4.502011	4.159289	1.434423	1.721906
GAPDH	1.126206	1.602765	2.102048	0.218574	3.601206	1.513021	4.150444	5.595125	0.121012	-0.04296
SMPD4	4.787213	2.307756	2.626378	2.211336	1.381125	4.017603	2.82263	3.763139	1.212535	1.968949
NUP107	2.81225	0.922905	3.225822	2.108469	2.878793	2.206825	3.269519	5.070968	0.258651	2.727392
CDK4	2.059662	1.772496	3.259721	2.164665	2.132514	2.829773	2.412833	4.344347	1.096989	1.431595
ARFGAP1	1.49218	1.815793	5.109728	1.647079	2.290461	2.252261	2.909427	1.3544	1.144427	1.298048
LMNB2	1.627505	1.96455	1.96663	1.201152	2.300771	2.93827	2.709408	3.692788	0.827485	1.84503
NUP62	1.737482	2.150971	2.005209	1.838025	1.38458	1.222131	2.752333	3.740428	0.804707	2.046537
DHX37	1.940277	0.992088	2.240988	0.952585	3.37774	2.214161	2.208455	2.003102	0.540926	1.676701
BRIP1	1.543909	1.66112	1.738025	0.820192	1.379549	0.891235	3.787494	4.148418	0.032005	1.255006
CEP131	2.166839	0.932255	1.544204	1.760097	1.876353	3.808749	1.572707	1.309156	1.053777	1.57551
CCDC34	1.54747	0.415754	2.343049	1.037374	1.318879	2.197729	2.011257	3.851062	0.596056	1.471107
AAAS	2.0406	0.81145	2.17886	0.89533	2.37432	1.65036	1.92246	3.21113	1.1638	0.51386
DTX2	0.77156	1.51259	1.789443	0.012217	2.925166	1.313978	3.011319	2.472488	0.013628	0.350852
LMNB1	1.244626	2.434563	0.845554	0.865485	1.372618	1.674689	3.013195	3.430106	1.03619	1.037406
STX1A	1.00897	1.955136	1.977488	2.149286	1.64814	2.04799	5.216325	3.033826	-0.03802	0.459864
ZC3HC1	1.304542	0.161291	1.386902	0.508866	4.551955	1.202404	2.507915	2.81661	0.151474	0.455195
NUP155	1.786093	0.764152	2.718671	1.569325	-1.54281	1.458431	5.02583	6.236842	-0.16727	1.896058
LEMD2	2.374107	1.347407	0.46335	1.261895	2.003261	3.37608	1.473642	1.793214	1.457569	0.536374
JPT1	1.195053	3.024425	-0.16704	0.62575	1.374603	2.377637	3.793235	4.598367	0.580001	0.390257
CPNE1	1.585547	1.063456	3.980296	1.534828	1.425698	1.342175	1.363162	1.745948	0.004133	2.621497
MRPS23	1.92583	3.385073	1.918327	1.42358	2.904453	3.175894	1.804378	2.048941	-0.05515	0.776687
NOPCHAP1	0.858263	0.629743	2.568475	0.543825	2.780682	0.982652	2.063694	2.080779	0.778768	0.122899
RTEL1	1.492555	0.938505	1.844561	0.932625	1.86398	2.374587	1.740015	0.830896	0.680986	1.52262
MRTO4	1.441702	0.867098	1.980582	0.640572	1.732194	1.341543	2.364338	2.757653	1.259736	1.852002
RANGAP1	1.105716	1.694025	2.536488	0.802401	-0.44436	0.827991	2.461789	4.429886	1.322358	1.067409
GTPBP4	0.610748	2.147087	3.451369	0.764323	0.783211	1.270621	2.842967	2.787087	1.268	3.539153
MFSD10	1.300259	1.391818	2.767726	1.354971	1.517324	1.686507	1.020797	-0.59336	1.038645	1.115766
CCDC77	1.688626	1.015451	2.1175	1.718692	1.313636	1.97778	1.544196	2.692547	0.776755	1.468343
XPO1	1.154854	0.741741	2.522005	1.093658	2.282029	1.601407	2.479854	3.277576	0.027093	1.715739
KPNB1	0.647557	0.974623	1.23107	0.660947	1.245548	1.112311	3.157564	3.637587	1.629554	1.197394
CMSS1	1.143448	0.75849	3.221954	1.740228	0.533084	2.125006	1.840985	4.956609	0.668352	1.079284
AEN	-0.55607	0.607667	2.486804	0.500007	2.978621	0.595137	0.684383	0.455035	0.472485	1.120366
TEPSIN	1.631336	0.709726	1.493065	1.255133	1.510952	2.503353	1.425977	1.462409	0.674482	1.126957
SLC16A3	0.947795	2.054391	0.188203	0.963976	2.348924	1.163031	2.071332	0.927895	-0.81937	0.67642
LRR1	1.364814	1.592414	0.202094	1.543402	0.30185	1.522619	1.746366	2.745572	0.19556	0.850258
TMEM223	1.54838	1.59497	0.767762	0.935283	1.290367	0.48594	1.384105	1.715856	0.329336	0.37979
MAD1L1	1.240585	0.665951	0.829044	1.313913	3.560755	0.873276	0.62163	0.168394	1.080642	1.170867
CEMIP	0.844143	2.727593	6.351233	0.241236	-0.8562	1.195652	1.314631	0.769418	-0.8016	2.217983
SMOX	0.056914	0.438151	4.49591	0.784407	1.040695	0.591034	1.746624	2.563808	0.662236	0.080332
TXNL4A	1.003526	1.94337	-0.47327	-0.64475	0.968622	1.656264	1.193031	1.932246	0.895681	-0.06361
ATP5MF	1.469613	1.384266	0.477844	0.061248	2.049821	1.800509	1.171119	1.609018	0.869256	0.10536
BRAP	0.793854	-0.63106	0.180732	0.735558	2.080498	1.411603	1.358165	1.87021	-0.89877	1.233248
POM121	0.435686	-0.37624	1.966161	0.564493	1.666117	0.507428	0.659439	1.695016	1.274434	0.99303
NDUFB4	0.878928	1.413223	-0.40952	-0.01667	1.704938	0.371531	1.462013	2.99648	0.813682	-0.77724
TMEM201	2.03406	0.49537	2.378493	0.2502	-1.43018	3.29468	1.210803	2.988086	0.760926	2.113463
RBMX2	0.326401	0.41108	2.637282	1.065504	1.15554	1.40804	1.045467	1.898071	0.170305	1.038277
REXO5	0.454298	1.095735	1.678229	0.068882	1.107567	1.910898	1.877335	2.298116	0.360615	0.678397
C5ORF22	1.491573	1.021637	-0.01067	0.487656	-1.47547	1.019234	3.427069	5.306831	0.145301	1.221656
RIN1	0.641789	0.115114	2.246789	0.562469	2.9645	0.434172	0.906535	1.598969	-1.08956	0.673885
UTP18	0.811875	1.400552	2.524457	0.826388	1.241678	1.477996	1.125472	2.603383	0.869738	1.685954
TMEM209	1.298008	1.223392	1.051957	1.293396	1.080584	1.774461	1.80949	1.549469	0.15336	1.740761
ZBTB9	0.876719	1.060389	2.830413	1.08035	-0.04537	2.479754	2.115361	2.257708	0.855227	1.274424
NRM	1.358482	1.265212	1.041438	2.233432	2.04008	2.355328	0.892802	2.694421	-1.28295	1.276334
TMPO	1.624991	0.991992	2.010433	0.691903	-0.79562	0.534914	2.435694	2.87605	-0.17908	1.601067
TIGD3	0.916022	2.038204	1.318236	1.037951	0.071214	1.770852	1.833079	1.530663	0.89316	0.406077
POM121C	0.303716	-0.4424	1.739394	0.641847	0.937198	0.663501	0.630997	1.63335	0.93914	0.941674
SNAPC4	0.866817	0.622094	2.46956	0.900884	0.544443	0.546236	0.5749	0.476613	1.777085	1.685053
NDC1	1.595964	0.805649	1.238607	0.812607	-2.08212	0.766219	3.579344	4.396332	0.350505	1.743769

TFDP1	1.631158	1.870209	3.471224	0.530195	-1.09159	-0.34907	2.763548	5.960794	0.045468	1.898871
KIAA0930	-0.70389	0.47231	1.275721	1.40461	3.108573	-0.0241	0.191701	2.151897	-0.56524	0.905932
ZBED10P	1.391363	0.511938	1.490793	0.397141	1.38458	1.34641	1.199823	0.328097	-0.27223	1.307764
KLK6	-0.08009	-1.85639	10.28156	-0.48848	-1.99911	-1.58743	1.967696	3.996846	-0.70549	0.643427
CREB3L4	1.226778	1.358247	0.068258	-0.61627	1.160282	2.59408	3.181758	1.958532	0.787259	0.07741
AARSD1	-0.34746	0.196686	0.56179	-0.34341	3.279352	1.320518	3.522051	2.736296	0.713939	-0.17074
GPR37	-0.60928	0.392837	-0.36518	-0.7415	0.70801	-0.97065	2.392702	1.267284	1.115832	-0.33472
FZR1	0.826579	0.856885	1.336588	0.715959	-0.28582	0.533071	0.948901	1.887664	-0.06035	-0.1903
ZDHHC16	1.298944	2.242896	0.808425	0.743148	1.602215	-0.35269	1.508866	-0.37117	0.21183	0.407221
GTF3C3	0.339323	-0.77062	2.682717	0.569938	0.840366	1.062166	2.024934	3.718285	-0.38847	1.255643
NANP	0.882663	0.405289	3.194912	0.908597	0.037996	0.640581	1.277586	2.333295	-0.41039	1.512798
INTS14	0.795189	1.095306	1.241555	0.974182	-1.4249	-0.79943	1.596158	3.135002	0.239978	1.442036
RETREG3	0.969195	0.52855	-0.51613	0.501317	2.971171	0.436615	1.104064	0.846457	0.208711	0.04098
YEATS4	2.188083	1.06692	1.639907	0.657466	-0.7406	0.439839	0.902995	2.285376	0.57754	1.236894
NUP50	0.786219	0.693223	0.334324	0.545629	0.073816	-0.3214	1.242955	3.493386	0.124285	0.697221
TENT4A	0.801634	-0.83058	1.960455	1.304871	0.171771	0.146909	0.625217	2.027969	0.234198	0.934828
ARL6IP6	0.881998	0.264711	1.343049	1.036248	1.209567	0.963095	0.398118	2.451772	-0.58601	1.193565
CERS5	0.489749	0.530945	1.052475	1.572033	0.66341	1.691491	0.768629	1.805806	-0.48243	0.659286
ZBTB41	0.416655	1.14578	1.374811	0.438731	-0.70037	1.518658	2.360196	1.657406	-0.15075	1.43351
BLTP3A	0.815293	0.322554	1.25836	0.372928	-0.03575	1.02823	1.088754	0.306582	0.905885	1.189157
KIAA1549	0.275887	0.441562	4.248161	0.014588	-0.33552	0.732147	0.717912	0.846743	0.208271	1.379551
GLE1	0.547714	2.027005	0.275356	0.989752	-0.29084	0.157306	0.615283	1.242249	0.487956	1.094904
MAD2L1BP	0.792585	2.053455	-0.63922	0.454347	0.244969	0.654215	1.373005	1.05361	0.29796	0.228537
C2ORF68	1.676407	-0.15024	-0.50362	-0.0364	1.163976	1.596645	1.733471	1.793752	0.907816	0.15165
THAP7	0.760773	0.97151	0.575744	0.366768	0.102453	0.258449	0.688051	1.66801	0.063845	-0.60394
METRN	0.117334	2.289709	-0.05427	0.44385	1.884092	-0.71808	0.373314	-0.59875	0.174028	-0.35554
AMPD3	0.021698	0.229193	0.527077	0.150642	-1.44083	0.234145	1.187864	0.601966	0.394396	0.139937
PROCA1	1.111906	0.311388	1.078021	0.230223	1.485476	1.527509	0.580516	-0.51293	-0.44363	0.573329
NUP35	0.369093	0.030626	1.872923	0.170299	0.649876	0.117309	1.941405	2.432636	0.294066	1.404975
CERS2	1.682395	3.740149	0.860238	1.458649	0.759369	1.181692	-0.14942	-2.24186	1.370781	0.727836
DCTN5	-0.12897	2.352978	0.586708	0.365741	0.074179	0.618286	1.456296	2.138532	0.142258	-0.02646
SUN1	0.663662	-1.7368	1.116087	0.26488	1.553279	1.411358	1.198806	2.09598	0.321776	0.414191
EBP	1.251901	1.973464	1.198842	0.197139	-0.65543	-0.85439	0.111421	0.968504	-0.3597	0.178141
AHCTF1	0.423338	0.683973	1.894304	0.884396	-2.12642	1.410741	1.732	2.500071	-0.20372	1.282555
EMD	-0.35716	1.490628	2.95673	0.213499	0.011016	1.46574	0.894895	1.48201	0.431099	-0.47176
SIX2	0.252468	1.356694	1.299279	-0.12283	-0.7739	4.842753	1.602714	2.644594	-0.6829	-0.35199
TNRC18	-0.36676	0.87538	0.856903	1.039271	-0.43692	1.761629	0.698878	0.243179	0.325989	0.719917
ECEL1	1.523885	-0.15478	0.733656	0.266151	-1.53108	0.609489	2.895466	1.356829	0.410438	0.805143
TNFAIP2	0.772779	-0.30415	-0.03689	-0.03856	2.159715	0.140204	0.338298	0.21838	-0.0784	1.10808
LFNG	0.321245	0.574874	1.375416	0.785934	1.324057	0.437572	1.479924	-1.3927	0.941468	0.722402
STX3	1.064411	1.252237	-0.71805	0.502483	0.994784	0.4012	-1.65296	-3.14265	0.282032	0.58603
QRICH2	0.986577	-0.01317	0.500543	-0.90357	0.685214	1.599724	-0.12223	0.292636	1.421898	0.52367
SEC11A	0.852044	-0.25529	-0.78691	0.638072	0.801625	0.883382	-0.07598	-0.11634	0.223763	0.850212
SURF4	1.301824	0.368496	0.407172	0.997608	-1.56243	-1.07641	2.08274	0.726307	0.518628	0.185749
HEY1	0.748645	-1.42327	-1.93756	0.69615	-1.38611	1.604901	-1.35114	0.605474	-0.3895	0.682827
SUMO1	1.022632	1.368608	0.505704	0.911812	-0.80357	-0.5566	1.17722	1.293669	0.187014	0.764454
SMIM13	0.359061	0.4121	1.210467	0.563883	-0.64283	0.544306	0.03541	2.377469	-0.10021	0.705196
RANBP17	0.85274	0.81386	2.685931	-0.23589	1.495793	0.441324	-0.12063	-2.10663	1.402932	1.002811
ARMCX6	0.415008	-0.05354	0.533257	-0.77553	-0.1384	-0.94111	0.366184	1.118508	-0.29583	-0.72864
KCNC1	-1.00555	-0.5623	-1.81571	-0.73935	0.488251	3.476432	-0.28812	1.553316	-0.28481	-0.72145
NUP133	-0.09693	0.95018	1.81013	0.520372	-2.16787	2.693249	1.601964	2.41679	-0.68983	1.018955
KLK7	-0.33572	-2.35805	14.09072	-0.50718	-2.78241	-1.87042	-1.16398	-0.03882	-0.60415	0.092011
TPR	0.131891	0.184088	0.64114	0.349445	-0.59524	1.463356	0.851979	0.485943	-0.31731	0.967643
C6ORF141	0.03438	0.391192	1.013323	1.786309	0.910002	-0.66072	1.392156	1.978921	-0.65122	-0.05696
INPP4A	-0.48741	-0.41709	-1.21773	1.244063	-0.74435	0.465109	0.233822	-0.70906	-0.20803	0.053984
CADM4	0.271084	0.614325	-0.13357	-0.70912	0.140939	-0.68143	2.495286	3.664726	-0.24673	-0.80438
TMEM205	-0.03012	1.449268	1.695571	-0.45096	0.930786	-1.07204	0.624948	-1.14142	0.49221	-0.20234
MRPS14	1.49564	1.831697	0.277694	0.083817	-0.42553	2.048467	1.321077	1.516002	0.262843	-0.13722
KPNA4	-0.63734	0.055826	0.30087	0.332731	-0.64425	0.26207	-0.0725	2.768274	-0.81769	0.335662
SEC11C	0.406185	0.921715	-1.05291	-0.70933	-1.72487	0.76454	1.128475	-0.31946	0.502519	-0.3974
ICAM3	-0.02383	0.564115	-1.66873	-0.24385	0.462102	-0.99098	0.019216	-1.61428	0.964284	-0.29124

MX1	0.616102	1.277241	-0.24614	0.995883	-0.41366	-0.23289	0.259413	-0.67953	-0.73397	0.029006
UPK3A	-0.04697	-0.17817	0.345103	-0.87932	0.429935	1.87875	2.461304	-0.55629	0.072235	0.880542
TOR1A	0.355844	1.144626	-0.43083	1.880681	-0.30751	-2.81025	0.239116	0.350593	0.566149	0.397407
ADGRG5	-0.60586	-0.60365	0.107238	-0.24866	0.697415	-0.00905	0.638917	-0.56613	-0.76468	0.504487
RB1CC1	-0.18636	-0.36769	0.550868	0.244489	-1.03867	0.54059	-0.41006	0.810347	-0.17453	0.336559
CDIPT	0.188073	0.371173	-0.68736	0.814834	0.955034	0.014662	-0.62078	-0.37193	0.030166	-0.54965
RAP1GAP2	0.527306	-0.2542	-0.09057	-0.10567	-0.16537	-0.10257	0.76899	-0.26303	0.90538	0.386489
SIPA1L2	-1.83223	0.56293	-4.81891	-0.79988	-0.8311	0.871116	-0.40122	0.791136	-0.03193	-0.12679
LBR	-0.73575	-0.663	1.371759	0.612589	1.8616	1.14235	2.086926	2.29994	-0.35397	0.50733
RFX7	0.241813	-0.52351	0.941141	-0.42444	0.160488	0.371901	-0.20169	1.039255	-0.57022	0.770842
TET1	0.242441	-0.56209	-0.09077	0.423382	-0.75511	1.144784	1.390487	1.864845	0.052711	-0.10646
MLX	1.390668	-0.52807	-1.07301	-0.32786	0.324839	0.036967	0.076336	-0.45413	0.729457	-0.24065
MCM3AP	0.094538	0.263619	0.055064	-0.26238	-1.00367	0.867357	0.488641	-1.22504	1.394153	0.508019
PHF20	-0.58247	0.44812	1.898327	0.196011	0.622526	0.459957	-0.48902	-1.19674	-0.01242	0.550263
TMEM175	0.743496	0.239483	-1.74098	-0.4402	0.467877	-0.25727	0.269483	-2.69317	0.996295	-1.1441
KIAA1586	0.088664	-1.19837	0.538573	0.606919	0.328632	1.195464	0.514962	1.023889	-0.05069	-0.02966
TMEM154	0.256902	-0.05693	0.994942	-0.20544	0.281299	-1.67404	0.206508	1.389953	-1.55289	-0.29331
ZBED1	0.325082	0.213996	-0.93105	0.165682	-1.95344	-2.03869	1.726025	1.162344	0.839997	0.453402
FSTL4	-0.24859	0.841045	-0.2863	0.035323	-5.61799	2.600511	0.94835	1.314081	-0.13563	-0.48405
FA2H	-0.0669	0.409339	-0.84336	-0.65379	-1.06099	-0.6493	3.022975	-0.62397	0.324101	0.130905
LEMD3	-0.07093	-0.75076	-0.28554	0.542869	-1.52796	-0.76916	-0.84528	0.70583	-0.08858	1.053429
DNAJB2	-0.13868	0.490614	-1.18117	0.337081	-1.27826	1.472091	0.044431	-0.28019	0.192678	-1.12093
TNFSF15	0.087608	-0.35516	1.723268	-0.69856	-1.62664	0.480805	0.178459	-1.88473	-0.43989	0.834346
CLK1	-0.73551	-1.5306	0.690618	0.207139	0.942703	-0.18251	-0.01311	-0.53808	0.445174	-0.00718
LMNA	-0.87832	-0.27273	-0.54681	0.127573	0.707995	1.555578	-0.4253	-0.56249	-0.65508	-0.25775
TNKS	-0.67103	-1.13035	-0.05801	-0.34197	0.596147	0.28606	-0.0071	0.013856	-1.14267	0.844677
CCDC125	0.448662	0.881538	-0.23498	-1.68257	0.275399	-0.14078	0.898297	-1.36073	0.829036	0.009891
FAM166C	0.177172	0.247013	3.613604	-0.75878	1.518367	-1.38103	-0.15141	-0.36563	-0.41347	0.255731
POMZP3	0.006371	-0.67321	-0.25611	0.17556	-0.12025	0.259332	-0.33232	0.197766	-0.3647	-0.45021
NUP153	0.093826	-0.31429	1.125046	0.256577	-1.83413	-0.21272	0.203299	1.192179	-0.60065	0.890101
RNF6	0.569487	-0.32511	0.824628	-0.36331	-1.24639	-0.41552	-1.56188	-0.06983	-0.27244	1.161408
ANXA5	-0.95976	-0.84446	0.367713	1.013917	1.245269	0.440275	-1.3824	-3.8327	0.693478	0.641627
ZCCHC14	-1.09065	-1.19866	-0.01793	0.303068	0.650235	-1.21511	-0.22613	-0.71406	-0.68331	0.618707
STX1B	-0.65281	-1.59038	-0.61498	0.250243	1.816419	-1.07925	0.380605	0.058594	-0.29737	-0.11703
CACNA1D	-0.16899	0.597599	1.827084	-1.301	0.640228	0.244335	-1.5773	-6.6548	1.885861	1.475053
DUSP2	-0.54501	-0.1952	1.477183	-0.12558	-0.94778	-1.14221	-0.08637	0.157288	-0.68102	0.426491
CACNA1D2	-0.16899	0.597599	1.827084	-1.301	0.640228	0.244335	-1.5773	-6.6548	1.885861	1.475053
RNF63	0.569487	-0.32511	0.824628	-0.36331	-1.24639	-0.41552	-1.56188	-0.06983	-0.27244	1.161408
SAMD15	1.652977	0.727167	1.363295	0.258888	-0.79578	-0.33739	0.07015	-0.09953	0.122657	0.938721
FUT11	0.125227	-0.53552	-0.68715	0.278207	-0.13706	-0.45477	-1.06905	-1.21357	-0.23861	-0.03597
ANKRD17	-0.1764	-0.43045	-0.03069	-0.30095	-1.88096	-0.77666	0.191567	0.216218	0.047414	0.568602
DNAJB12	0.200653	0.049565	-0.79861	-0.76351	-0.6472	-0.19825	-1.79677	-3.26052	0.394944	-0.28471
RGPD1	-0.72649	-0.45352	-0.48917	0.211095	-1.20902	-1.20817	-0.11181	0.558725	0.224975	0.729215
NBAS	-0.81896	-1.38401	0.071952	-0.79327	-1.07154	1.240709	-0.08623	-0.40546	-0.79394	-0.06318
PLRG1	-1.00728	-1.82762	-0.46071	-0.42388	-1.35903	-0.08754	1.288219	1.920036	-0.8696	-0.30208
GCH1	-0.03897	1.08593	-0.43264	-0.08925	-1.78796	-3.3918	-0.10515	-0.38777	0.106084	0.507943
FAM169A	-0.12629	-0.02199	1.924411	0.339117	-5.39458	0.492924	0.729726	2.496737	-1.49396	0.660049
TTLL2	-1.21838	-0.66237	0.740846	-1.07202	1.623472	-1.66	0.521652	-1.09133	-0.19057	1.070247
ZBTB1	-0.1757	-1.33872	0.092111	0.624627	-0.34943	-0.69625	-0.49837	-0.75843	0.368237	0.596809
SLA	-0.84224	0.462667	-1.39901	0.556821	1.292115	-0.90592	-2.1669	-3.46922	0.082888	0.273837
ENDOD1	-0.92633	-2.26241	-3.75482	-1.41185	0.435049	-2.11331	0.63302	0.347136	0.469919	-0.5254
CCDC71	0.511475	0.233656	-0.36131	-1.05691	0.421636	0.325632	-0.29948	-1.38814	-0.05553	-0.93975
PHF11	-0.44976	-0.3458	0.829893	-0.01631	0.239631	-0.09538	-1.04071	-2.36532	-1.61005	0.127665
ATP6V1B1	1.544039	-0.34314	1.454828	-0.40178	-7.83999	0.61119	-0.31561	0.228677	-0.80297	0.737142
CAPN8	-0.75684	-0.00141	0.026668	-0.93328	-0.92899	0.789775	0.824967	-6.56988	-0.53495	0.281359
GDF6	-0.69717	-0.54656	-1.89553	1.014326	-1.64809	-1.5425	-0.53107	-0.17501	-0.50292	-0.36377
ABHD18	-0.07991	0.01015	-1.09483	-0.59526	-1.74709	-1.99853	1.280109	-0.35324	-0.17625	-0.38772
RGPD6	-0.76565	-1.94708	0.41423	-0.75562	-0.72577	-0.55781	-0.67254	-1.90126	0.101338	0.228172
CCDC136	-1.12365	-2.16686	-0.883	-0.07874	1.607539	0.213062	0.074926	0.893824	-0.90139	-0.29849
KLHL11	-0.46401	-0.41756	-0.50688	-0.15956	-1.05027	-0.64667	0.087599	0.525453	-0.59149	0.792994
FAM120C	-0.47313	-0.38957	0.71112	-0.81152	-1.62357	-0.32584	-0.25289	0.216448	-0.69147	0.026869

IPO5	-0.43063	-1.30188	2.695876	-0.5484	-3.01764	-0.10476	0.373637	0.854429	-0.85878	0.615737
RGPD4	-0.85524	-1.62437	-0.05826	-0.63165	-0.93164	-0.51713	-0.25739	-1.54293	0.701348	0.56423
LCORL	0.181416	-0.77967	0.277112	-0.40584	-1.17711	-0.93243	-1.4881	-2.83365	-0.15683	0.179702
NUDT9	-0.43072	1.079872	-2.69492	-0.81814	-1.10409	-1.16205	-1.7544	-2.75022	1.565287	-0.40484
DHRS4L2	-0.25314	-0.41659	-2.08312	-0.32799	-1.1494	-1.57056	-1.18155	-0.36055	-0.45165	0.467285
TMX4	-1.57527	-1.28747	-0.26532	-0.75287	-0.62925	0.372074	-0.55941	-2.30408	-0.78983	-0.48824
CES3	0.261822	-0.43853	-3.57219	-1.23626	-0.97689	-2.5157	-0.35026	-0.34662	-0.09585	-0.18341
SULT1E1	0.644098	-0.81521	-0.16401	0.404883	-1.74644	-1.93878	-1.02277	0.241327	-0.80415	0.03069
RGPD5	-0.62989	-1.51356	-0.85938	-0.34464	-1.19784	-0.20866	-0.57471	-0.90595	-0.7319	0.208073
SCAI	-0.70586	-0.84606	0.341193	-0.09919	-1.12615	0.057275	-2.37086	-2.30767	-0.2782	0.38319
RAMP1	-2.29062	1.645854	-0.53143	0.038145	-2.44148	0.613961	2.177212	0.254065	0.730982	-0.47543
CASC3	-0.76196	-0.96633	0.299964	0.072821	-2.62472	3.322213	-1.21373	-0.86121	-0.36575	0.263944
CYTL1	-2.03248	-1.75293	-1.76792	-0.42996	-0.09159	0.816508	-0.81654	-1.28513	-1.14326	-1.16412
CRY1	-0.39555	-0.34614	0.209374	-0.51347	-0.50242	-0.37873	-1.13698	-1.27737	0.133354	-0.46957
RGPD3	-0.65682	-1.60825	-0.05733	-0.61408	-2.44494	-0.74166	-0.53768	-1.43632	0.210419	0.089677
DHRS4	-0.41277	-0.17284	-1.87619	-0.39685	-1.18828	-2.2215	-1.39649	-0.50819	-0.61878	0.086655
RANBP2	-1.6081	-0.98774	0.661567	-0.72603	-2.40314	-1.02666	-0.32674	-0.48469	0.484643	0.527496
KLHDC2	-1.14408	-1.66172	-1.8707	-0.64589	-0.37845	-1.30107	0.458622	-0.65785	-0.42142	-1.05821
TBC1D19	-1.00747	-0.73386	0.202625	0.151373	-1.39432	0.688672	-0.89561	-1.48669	-0.98162	-0.55342
EOGT	-1.22988	-4.19159	0.121379	-0.33145	0.359622	0.838811	-2.81677	-2.89939	-1.98566	0.188248
TAF3	-0.31949	-0.55212	0.051457	0.233775	-2.90571	-0.41233	-1.18909	-1.61769	-1.3424	0.556596
SYNE2	-0.7652	-2.00147	-1.21465	-0.38567	-0.90015	-0.95173	-0.67819	-0.5998	-0.22823	0.678236
C11ORF42	-1.60869	-0.72768	-0.93729	-1.13374	-0.31155	-1.39289	-0.71773	-0.93566	-0.68958	0.136397
NHSL1	0.881754	-1.09066	-1.57034	-0.0349	-3.3277	-0.80505	-4.04944	-1.41902	1.195134	-0.0121
SHPRH	-0.46207	-1.2811	-0.2895	-0.52574	-2.40938	-0.42764	-0.74236	0.233288	-0.34975	0.201318
TIAM1	0.166088	0.563849	-4.06121	-0.33934	-1.70213	-2.93945	-1.63438	-0.13615	-0.6681	-0.5539
SDCBP	-1.56264	-0.8152	-0.86509	0.084939	1.046886	0.444036	-2.629	-4.71151	0.403436	0.183917
CYP2B6	-0.2389	0.469476	0.293748	-1.05189	-3.19787	-3.46273	-0.33377	-2.21866	-1.18534	0.576872
C22ORF31	-0.49451	-0.99562	-1.7967	-0.70424	-2.02909	-1.87272	-1.04436	0.065539	-0.95926	-0.99341
HAND1	-1.12672	-1.02902	-1.53647	-0.79507	-1.93595		-1.16996	-1.01786	-0.49267	0.615079
NAV3	-3.61723	-2.82488	-1.49054	0.374936	1.153354	0.823996	-1.38005	-2.2161	-0.97915	-0.02519
MCM3AP4	0.094538	0.263619	0.055064	-0.26238	-1.00367	0.867357	0.488641	-1.22504	1.394153	0.508019
SUN2	-1.28862	-1.80987	-2.12117	-0.76233	-2.41203	-2.49107	-2.28927	-1.04073	0.347183	-1.24643
PRNP	-1.23063	-4.26999	-0.89005	1.832682	-0.00841	-3.07169	-2.7848	1.842272	-1.57145	-0.66859
SLC5A1	-0.44344	-0.94459	0.493142	-2.02733	-0.47316	-6.73279	-3.21422	-2.21434	-0.1118	-0.04916
TMEM109	-0.51197	-0.92816	0.702692	-0.81054	-3.84156	1.145217	-3.15132	-1.40122	-0.66732	-0.68757
KDR	-0.85079	-1.87903	-0.15255	-0.01473	-4.98535	-0.94947	-1.64041	-4.04055	-0.80443	0.736124
AKIRIN1	-1.39527	-0.60668	-1.10725	0.267331	-3.83442	-1.33952	-0.19045	-0.27435	-1.07696	-0.51157
FAM135B	-1.6632	0.354426	-7.56122	-1.01114	0.639683	0.376284	-0.6771	-1.49246	-0.49861	-1.81859
SLC2A13	-0.92234	-0.47355	-4.45496	-0.64692	0.26725	-1.11264	-1.17305	-2.73778	-0.14912	-0.34459
BTNL9	-2.83379	-3.54183	0.633713	-0.72137	-2.50684	0.339614	-4.18025	-6.10302	0.080094	0.557266
HS6ST3	-0.23915	0.945509	-2.27684	-0.86362	-8.70928	-0.99992	-0.2358	-0.03088	0.602909	-1.61693
KIAA1614	-1.36197	-2.49144	-1.55461	0.458239	-2.11243	1.58817	-1.82876	-1.73173	-1.314	-0.84295
KCNJ13	-0.15067	-0.76888	-0.42069	-0.91383	-3.04598	-1.35258	-0.69473	-0.4979	-0.77119	-0.5328
STX2	-1.25259	-0.67742	0.380378	1.126977	0.985573	-0.08899	-2.89157	-5.15558	-0.82114	0.14337
FAM181B	0.060288	-1.29247	-3.27647	0.086052	-2.57829	-1.16364	-0.75552	4.456296	-1.08078	-1.6223
PAK5	-0.46325	-2.43054	-1.63373	-2.177	-2.12217	-2.37772	-1.93292	1.824209	-2.46165	-1.59331
ARHGAP1(	-1.44674	-2.67792	-1.28488	-0.68908	-0.00589	-1.84056	-1.65174	-3.16909	-1.1834	-0.87798
HABP4	-1.61058	-1.78779	0.432538	0.002437	-1.43259	0.795587	-1.17272	-3.053	-0.85082	-0.19812
CDH5	-1.43376	-1.9828	-0.64506	-0.10233	-6.56078	-0.53544	-4.09778	-6.71939	-0.13792	0.54273
DBX2	-1.47121	-2.39367	-1.71904	-0.76805	-1.3968	-5.41178	-2.06416	0.975954	-0.79384	-0.10958
HOXA7	-1.29102	-6.27105	-1.11705	0.715222	-1.61372	0.727656	-1.67593	-0.47205	-1.9882	-0.76956
SYNE3	-1.85808	-2.08874	-3.16604	-0.45172	-1.85564	0.738243	-3.20447	-4.6076	-0.36023	-0.66788
TOR1AIP1	-1.08626	-1.08398	-0.90079	-0.69172	-4.88867	-0.22468	-2.06786	-2.91672	-2.27187	-0.7656
SSPN	-1.76167	-4.06745	-2.40338	-0.47815	0.292063	-0.76735	-1.48297	-1.57802	-1.68504	-0.59283
ZC3H12C	-1.96654	-2.68623	-4.87993	0.102259	-1.22237	-1.5883	-2.38544	-2.03144	-0.79631	-0.93415
GRK5	-1.74505	-2.17626	-0.89352	0.38308	-2.21653	-0.81969	-5.24503	-5.71304	-0.8286	-0.47572
PCDHB4	-1.86403	-3.50644	-1.81843	-0.7295	-2.06842	-0.27956	-1.69106	-1.7502	-0.3168	-0.55341
MINDY3	-1.48265	-1.41558	-0.83877	-1.35683	-2.13448	-2.57908	-1.75828	-2.33464	0.494861	-0.77355
MPL	-1.44452	-2.97404	-1.45344	-0.97779	-0.1659	-0.3922	-1.38105	-2.44874	-1.16828	-0.5462
RADIL	-1.88642	-2.24801	-2.48199	-0.21663	0.114763	0.209427	-4.91879	-3.25026	0.18621	-1.32902

DIRAS3	-2.8563	-1.37048	-1.84302	-1.12997	-1.99222	-4.11587	-1.37109	-3.70456	-1.4409	-1.53743
ADPRH	-1.06235	-2.32515	-0.70832	-0.08804	-0.61591	-0.19282	-6.64436	-10.5286	-0.88201	0.057678
NTRK3	-2.7627	-3.11977	-2.94162	-1.14707	-2.76088	-1.4925	-2.98846	-4.58856	-0.99024	-1.18858
FOLR1	0.896875	-1.45521	2.276137	-0.77204	-3.52663	0.166445	-5.03716	-12.2418	-1.5219	-0.14206
TMEM47	-1.36458	-3.56855	-1.59921	-0.45061	-1.82768	-3.73287	-3.01385	-4.37611	-1.54601	-0.75492
MRGPRF	-3.26976	-2.84984	-1.63335	-0.06703	-6.05607	-2.05549	-1.95934	-2.26352	-1.18862	-0.85008
SYNE1	-2.5887	-2.87607	-2.57113	-0.62593	-3.89367	-2.91571	-2.28267	-7.41233	-0.80881	-0.59602
